# Molecular Mechanisms of Electroacupuncture-Induced Spinal Microglial Reprogramming in Neuropathic Pain: A Systematic Search–Narrative Review

**DOI:** 10.3390/brainsci16090914

**Published:** 2026-08-27

**Authors:** Boon Khai Teoh, Trang Thi Hoai Nguyen, Kotha Peddanna, Tran Van Bao Quach, Jaung-Geng Lin, Yi-Hung Chen

**Affiliations:** 1 Graduate Institute of Acupuncture Science, China Medical University, No. 91, Hsueh-Shih Road, Taichung 404328, Taiwan; pulau1959@gmail.com (B.K.T.); peddannakotha87@gmail.com (K.P.); 2 Graduate Institute of Chinese Medicine, China Medical University, No. 91, Hsueh-Shih Road, Taichung 404328, Taiwan; nthoaitrang@ctump.edu.vn; 3 Faculty of Traditional Medicine, Can Tho University of Medicine and Pharmacy, Can Tho 90000, Vietnam; 4 International Master Program in Integrative Health, China Medical University, Taichung 404328, Taiwan; 5 Chinese Medicine Research Center, China Medical University, Taichung 404328, Taiwan; 6 Department of Computer Science and Information Engineering, Asia University, Taichung 413305, Taiwan; 7 Office of Research & Development, Asia University, Taichung 413305, Taiwan

**Keywords:** electroacupuncture, neuropathic pain, microglia, neuroinflammation, neuroimmune modulation

## Abstract

**Highlights:**

**What are the main findings?**

- Across 26 preclinical studies, electroacupuncture modulated microglia-associated signaling pathways involved in neuropathic pain.
- Electroacupuncture suppressed pro-inflammatory microglial activation and promoted pro-resolution neuroimmune regulation.

**What are the implications of the main findings?**

- Microglial reprogramming may represent a key mechanism underlying electroacupuncture-induced analgesia.
- These pathways may guide future studies on optimized electroacupuncture parameters and translational biomarkers.

**Abstract:**

**Background:** Neuropathic pain (NP) is a debilitating chronic condition driven by maladaptive neuroimmune interactions in the spinal cord, with microglia playing a central pathological role. Electroacupuncture (EA) has shown analgesic effects in clinical and preclinical studies, but the microglia-centered mechanisms underlying these effects remain incompletely integrated. This review aims to provide a comprehensive mechanistic synthesis of how EA modulates microglia-associated neuroinflammatory pathways in neuropathic pain. **Methods:** This systematic search-narrative review used systematic search and screening procedures to identify English-language animal studies published between 2015 and 2025 in PubMed, CINAHL, Web of Science, and Cochrane Library. Twenty-six animal studies investigating EA effects on microglia-associated signaling pathways in neuropathic pain models met the inclusion criteria. Because of substantial heterogeneity in neuropathic pain models, EA parameters, molecular endpoints, and behavioral outcomes, findings were synthesized narratively, and no meta-analysis was performed. **Results:** The reviewed evidence revealed four convergent mechanistic categories through which EA modulates microglial activity: (1) attenuation of purinergic microglial activation via downregulation of IRF8, P2X4R, P2X7R; (2) suppression of innate immune sensing and inflammasome pathways, including TLR4/MyD88/NF-κB and NLRP3 signaling; (3) inhibition of downstream inflammatory amplification through p38 MAPK, PI3K/AKT, and COX-2 pathways; and (4) promotion of pro-resolution mechanisms involving IL-10/β-endorphin, PD-L1, GRK2/TREM2/DAP12, α7nAChR, GLP-1R, and GABAergic signaling. **Conclusions:** The available preclinical evidence suggests that EA modulates multiple microglia-associated pathways, attenuating inflammatory signaling while enhancing selected pro-resolution mechanisms. These findings provide a mechanistic framework for understanding EA-induced analgesia in neuropathic pain and highlight microglial signaling networks as important targets for future experimental and translational investigation.

## 1. Introduction

### 1.1. Neuropathic Pain and the Limits of Current Management

Neuropathic pain (NP) is officially described by the International Association for the Study of Pain (IASP) as pain caused by a lesion or disease affecting the somatosensory nervous system [1]. Current management follows an individualized, stepwise, and multimodal approach. Tricyclic antidepressants, serotonin–noradrenaline reuptake inhibitors, pregabalin, and gabapentin are commonly recommended as first-line pharmacological treatments, whereas topical lidocaine or capsaicin and tramadol may be considered in selected patients. Strong opioids are generally reserved for later-line use because of concerns regarding adverse effects, tolerance, dependence, and uncertain long-term benefit [2,3]. Broader treatment algorithms additionally emphasize early multidisciplinary care, including rehabilitation, psychological support, exercise, and sleep management, followed where necessary by combination pharmacotherapy, specialist interventions, or neurostimulation [4]. However, treatment effects are generally modest and variable [5,6], and many patients do not achieve clinically meaningful pain relief [7]. These limitations support the investigation of complementary strategies capable of modulating multiple mechanisms involved in NP.

### 1.2. Microglia in Neuropathic Pain

Recent neurobiological investigations have shifted the focus from purely neuronal mechanisms toward the pivotal role of the central nervous system (CNS) immune environment [8]. Within this context, microglia—the primary resident immune cells of the CNS—have emerged as key contributors to the pathogenesis and chronicity of NP [9,10]. Following peripheral nerve injury, persistent afferent activity induces hyperexcitability in dorsal-horn nociceptive circuits, while signals from injured and hyperactive neurons recruit spinal microglia. Activated microglia, in turn, release pronociceptive mediators that enhance excitatory transmission and weaken inhibitory control. Central sensitization and microglial activation therefore form a bidirectional feed-forward loop in which neuronal hyperactivity promotes microglial reactivity and microglial signaling further reinforces neuronal sensitization [8,11].

Under physiological conditions, microglia exist in a ramified state, maintaining tissue homeostasis [12]. Following nerve injury, they undergo dynamic changes in morphology, gene expression, metabolism, phagocytic activity, and cytokine release [13]. These responses have traditionally been described using the M1/M2 framework [14,15]. The pro-inflammatory M1-like phenotype is characterized by the release of cytotoxic mediators, including tumor necrosis factor-α (TNF-α), interleukin-1β (IL-1β), and reactive oxygen species (ROS), which collectively enhance the excitability of dorsal horn neurons and exacerbate pain symptoms [16,17]. Conversely, the neuroprotective M2-like phenotype releases anti-inflammatory cytokines, such as interleukin-10 (IL-10), to resolve inflammation and promote tissue repair [14,15]. The disruption of the dynamic equilibrium between these two states is a hallmark of NP, driving the transition from acute to chronic pain [18]. Although the M1/M2 terminology is useful for summarizing pro-inflammatory and pro-resolution tendencies, microglial phenotypes in vivo exist along a dynamic spectrum rather than as fixed binary states [13,19].

Peripheral nerve injury initiates several interconnected neuron–microglia signaling pathways. Injured sensory neurons upregulate colony-stimulating factor 1 (CSF1) and transport it to their central terminals in the spinal dorsal horn, where it binds to CSF1 receptor (CSF1R) expressed by microglia. CSF1–CSF1R signaling promotes microglial proliferation and induces a DNAX-activating protein of 12 kDa (DAP12)-dependent transcriptional program involving interferon regulatory factor 8 (IRF8) and interferon regulatory factor 5 (IRF5) [20,21,22,23,24]. The IRF8–IRF5 axis promotes the expression of purinergic P2X4 receptor (P2X4R), thereby increasing microglial responsiveness to extracellular adenosine triphosphate (ATP). Subsequent activation of P2X4R stimulates the release of brain-derived neurotrophic factor (BDNF) from microglia [25]. Microglial BDNF then activates tropomyosin receptor kinase B (TrkB) on dorsal-horn neurons, reduces the expression and function of potassium–chloride cotransporter 2 (KCC2), and disrupts neuronal chloride homeostasis. The resulting impairment of γ-aminobutyric acid (GABA)-mediated and glycinergic inhibition contributes to neuronal disinhibition, tactile allodynia, and central sensitization [25,26].

Following neuronal injury, stressed sensory neurons and, in some pathological conditions, central neurons may upregulate and secrete C-C motif chemokine ligand 2 (CCL2), also known as monocyte chemoattractant protein-1 (MCP-1) [27,28]. CCL2 signaling through its principal receptor (CCR2) is particularly important for the recruitment of peripheral monocytes and macrophages, although direct effects on resident microglia have also been reported in selected experimental models [29]. Chemokine-receptor signaling can activate intracellular cascades including p38 mitogen-activated protein kinase (p38 MAPK), extracellular signal-regulated kinase (ERK), phosphoinositide 3-kinase (PI3K), protein kinase B (Akt), and nuclear factor kappa B (NF-κB) [28,30,31,32]. Activation of these pathways also stimulates P2X4R expression and amplifies pain signaling [33]. Separately, Akt–mechanistic target of rapamycin (mTOR) signaling has been associated with altered autophagic activity and pain hypersensitivity in some peripheral nerve-injury models [34,35,36]. However, a direct CCL2–CCR2–PI3K/Akt–mTOR pathway that suppresses autophagy specifically in microglia has not been established. The contribution of mTOR to neuropathic pain appears to be dependent on cell type, injury model, and disease stage.

Microglia also respond to damage-associated molecular patterns (DAMPs) released or exposed by injured and stressed cells. These signals include high-mobility group box 1 (HMGB1), S100 proteins, extracellular histones released from necrotic cells, heat-shock proteins (HSPs), mitochondrial components, and extracellular ATP [37,38]. HMGB1 and related DAMPs can activate pattern-recognition receptors, including Toll-like receptor 2 (TLR2), and Toll-like receptor 4 (TLR4) [39]. TLR4 recruits MyD88 and activates NF-κB-dependent inflammatory transcription [40,41,42]. In parallel, extracellular ATP activates purinergic P2X7 receptor (P2X7R), inducing potassium efflux and facilitating the assembly of the NOD-, leucine-rich repeat-, and pyrin domain-containing protein 3 (NLRP3) inflammasome. This process promotes caspase-1 activation and the maturation of IL-1β and IL-18 [37,43,44,45]. Triggering receptor expressed on myeloid cells 2 (TREM2), through its signaling adaptor DAP12, functions as a context-dependent injury-sensing and state-modulating receptor. In neuropathic-pain models, increased TREM2–DAP12 signaling in spinal microglia may promote inflammatory cytokine production and reinforce reactive microglial states [20]. Reactivated microglia release TNF-α, IL-1β, IL-6, BDNF, nitric oxide, and reactive oxygen species, thereby increasing glutamatergic transmission, facilitating N-methyl-D-aspartate (NMDA) and α-amino-3-hydroxy-5-methyl-4-isoxazolepropionic acid receptor (AMPAR) activity, impairing GABAergic and glycinergic inhibition, and promoting long-term maladaptive plasticity within spinal nociceptive circuits [11,24,46,47,48,49,50]. In addition to ionotropic P2X receptors, microglial P2Y12 receptor (P2Y12R) mediates process extension, chemotaxis, and p38 mitogen-activated protein kinase (p38 MAPK) activation after nerve injury [51,52]. Inhibition of P2Y12R reduces spinal microglial activation, excitatory synaptic transmission, and neuropathic pain behaviors [53]. P2X7R activation also drives p38 MAPK-dependent release of the lysosomal protease cathepsin S (CTSS) from microglia. Extracellular CTSS cleaves membrane-bound fractalkine (FKN) on dorsal-horn neurons and astrocytes, and soluble FKN then engages microglial CX3C chemokine receptor 1 (CX3CR1) to sustain p38 MAPK activation and TNF-α production [54,55]. This feed-forward loop prolongs microglial reactivity and helps maintain the chronic neuroinflammatory state of persistent NP. Thus, microglia contribute not only to the initiation of neuropathic hypersensitivity but also to its maintenance through reciprocal neuron–glia signaling.

**Figure 1:**
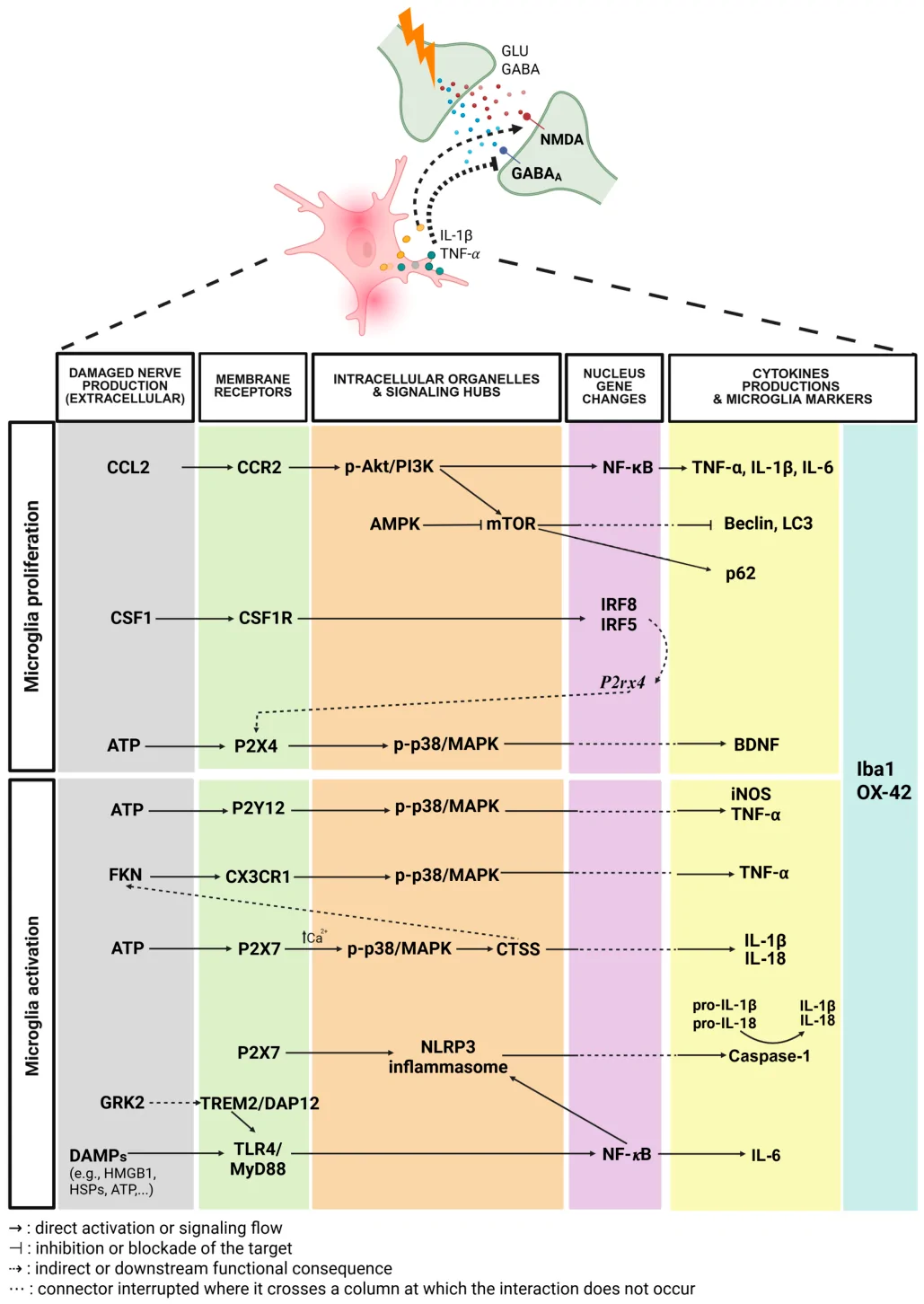
Microglia-centered signaling cascade in neuropathic pain. Peripheral nerve injury activates spinal microglia through neuronal danger signals, ATP, chemokines, and DAMPs (representative examples: HMGB1, HSPs, ATP). These signals engage CSF1R, purinergic receptors, TLR4/MyD88, TREM2/DAP12, PI3K/AKT, NF-κB, NLRP3, and p38 MAPK pathways, leading to the release of pro-inflammatory mediators such as IL-1β and TNF-α (yellow and green dots at the microglial surface), together with IL-6, ROS, NO, BDNF, CTSS, MMP-2/9, and COX-2. These mediators facilitate NMDA-mediated excitation by glutamate (GLU) and impair GABAᴀ-mediated inhibition by GABA (red and blue dots in the synaptic cleft), promoting central sensitization and thereby sustaining neuropathic pain. Line styles and arrowheads are defined in the key. Vertical panels correspond to the levels named in the column headers, with the microglial markers Iba1 and OX-42 at the right; horizontal bands separate microglial proliferation (upper) from activation (lower). Connectors are routed for clarity, and colors group items visually without quantitative meaning. Gene names are italicized (*P2rx4*); all other labels denote proteins or small molecules. Akt, protein kinase B; AMPK, AMP-activated protein kinase; ATP, adenosine triphosphate; BDNF, brain-derived neurotrophic factor; Ca^2+^, calcium ion; caspase-1, cysteine-aspartic protease 1; CCL, C-C motif chemokine ligand; CCR, C-C motif chemokine receptor; CNS, central nervous system; COX-2, cyclooxygenase-2; CSF1, colony-stimulating factor 1; CSF1R, colony-stimulating factor 1 receptor; CTSS, cathepsin S; CX3CR1, CX3C chemokine receptor 1; DAMPs, damage-associated molecular patterns; DAP12, DNAX-activating protein of 12 kDa; FKN, fractalkine; GABA, γ-aminobutyric acid; GABAᴀ, γ-aminobutyric acid type A receptor; GLU, glutamate; GRK2, G protein-coupled receptor kinase 2; HMGB1, high-mobility group box 1; HSPs, heat-shock proteins; Iba1, ionized calcium-binding adapter molecule 1; IL, interleukin; iNOS, inducible nitric oxide synthase; IRF, interferon regulatory factor; LC3, microtubule-associated protein 1 light chain 3; MAPK, mitogen-activated protein kinase; MMP, matrix metalloproteinase; mTOR, mammalian target of rapamycin; MyD88, myeloid differentiation primary response 88; NF-κB, nuclear factor kappa B; NLRP3, NLR family pyrin domain containing 3; NMDA, N-methyl-D-aspartate receptor; NO, nitric oxide; OX-42, CD11b/c microglial marker; p-, phosphorylated; p62, sequestosome-1; *P2rx4*, purinergic receptor P2X4 gene; P2X4, P2X7 and P2Y12, purinergic receptors; PI3K, phosphoinositide 3-kinase; ROS, reactive oxygen species; TLR4, Toll-like receptor 4; TNF-α, tumor necrosis factor-α; TREM2, triggering receptor expressed on myeloid cells 2.

The spinal cord also contains endogenous mechanisms that restrain neuroinflammation and restore inhibitory control. IL-10 can suppress inflammatory mediator production and promote microglial β-endorphin expression, thereby linking immune regulation to endogenous opioid analgesia [56,57]. Cholinergic signaling through α7 nicotinic acetylcholine receptors may likewise limit cytokine release and glial activation [58,59]. Other regulatory pathways involving PD-1/PD-L1, GLP-1/GLP-1R, GRK2, miR-124, and TREM2/DAP12 may shift microglia-associated signaling toward a less inflammatory and more pro-resolution profile. Restoration of GABAergic inhibition further counteracts microglia-associated neuronal disinhibition, although this effect occurs primarily at the neuronal level. Collectively, these mechanisms describe a progression from injury-related microglial activation and danger sensing to inflammatory amplification, neuron–glia sensitisation, and endogenous counter-regulation. These interconnected processes are summarised in Figure 1.

### 1.3. Electroacupuncture as a Multi-Target Intervention

Acupuncture involves the stimulation of specific locations of the body (acupoints) by penetration with thin metal needles and their subsequent manipulation by manual, electrical or other forms of stimulation [60]. This traditional Chinese therapeutic technique has gained widespread recognition in Western medicine for its clinical efficacy in managing various pain conditions [61,62,63,64]. Electroacupuncture (EA), a modern modification utilizing controlled electrical currents, has demonstrated significant analgesic effects in heterogeneous NP models [65]. EA may offer a complementary strategy for neuropathic pain by engaging multi-level neuromodulatory mechanisms that are not limited to a single pharmacological receptor target, regulating nociceptive neurotransmitters, receptors, and glial cell activity at the central level [66,67]. Crucially, the modulatory effects of EA are parameter-specific; different electrical frequencies and acupoint selections activate distinct components of the microglial signaling cascade [67,68,69]. These multi-target effects may help explain why EA shows analgesic benefits across heterogeneous neuropathic pain models.

### 1.4. Aim, Scope, and Organizing Framework

Although growing evidence supports the analgesic effects of EA in NP, its microglia-associated mechanisms remain insufficiently integrated. This review provides a narrative synthesis of animal studies identified through a systematic literature search, focusing on how EA modulates these pathways. Guided by established models of microglial activation, neuron–microglia signaling, inflammatory amplification, and pain resolution [46], the included studies were organized into four mechanistic categories: initiation and purinergic activation; innate immune and inflammasome signaling; downstream inflammatory amplification and neuron–glia sensitization; and pro-resolution neuroimmune modulation. These categories were refined according to the principal molecular targets examined and are intended as a practical organizing framework rather than an established classification or temporal sequence.

## 2. Methods: Systematic Search, Study Selection, and Risk-of-Bias Appraisal

### 2.1. Literature Search and Study Selection

This review used a systematic search and screening process to identify preclinical animal studies investigating EA, microglia-associated mechanisms, and neuropathic pain. PubMed, CINAHL, Web of Science, and the Cochrane Library were searched on 22 November 2025. The search combined controlled vocabulary and free-text terms representing three concepts: EA, microglia or microglial activation, and neuropathic pain. Search terms within each concept were combined with “OR,” and the three concepts were combined with “AND.” The complete database-specific strategies are provided in Table S1.

The search was restricted to English-language articles published from January 2015 to November 2025 to provide a focused synthesis of contemporary preclinical evidence. Earlier studies were used to establish the conceptual background but were not included in the structured synthesis. Reference lists of eligible articles were also examined for additional studies.

Studies were eligible when they: (1) were original animal experiments using an established neuropathic pain model; (2) evaluated EA as the principal intervention; (3) assessed microglial activation, phenotype-associated markers, inflammatory mediators, or microglia-related signaling pathways; and (4) reported pain-related behavioral outcomes. Reviews, editorials, conference abstracts, clinical studies, non-English publications, reports without accessible full text, and studies that did not investigate EA-related microglial mechanisms were excluded.

**Figure 2:**
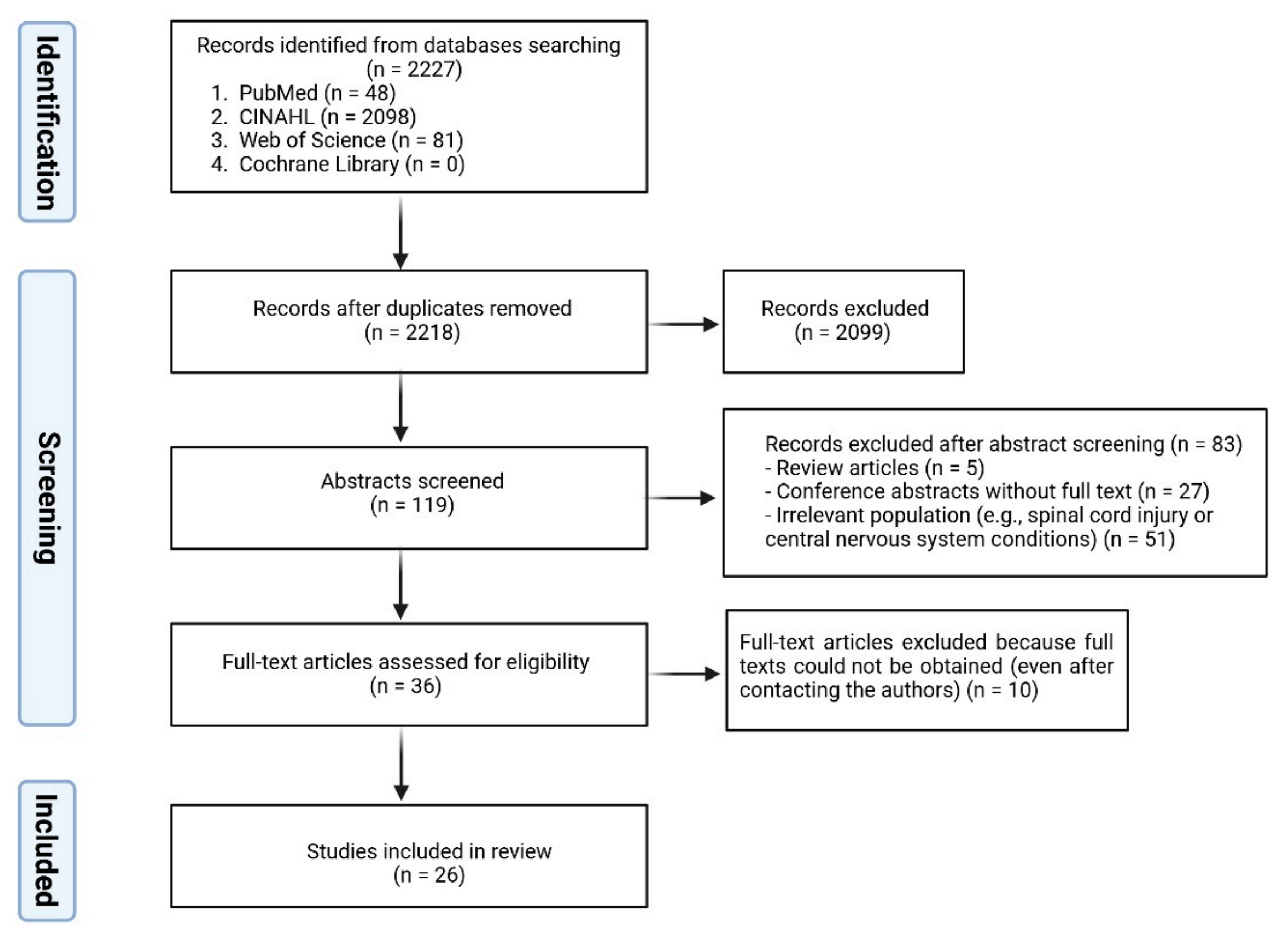
Flow diagram of study identification, screening, eligibility assessment, and inclusion. CINAHL, Cumulative Index to Nursing and Allied Health Literature.

Two reviewers independently screened titles, abstracts, and full texts. Disagreements were resolved through discussion, with consultation of a third reviewer when necessary. Data extracted from each study included animal species, strain and sex; neuropathic pain model; EA acupoints, frequency, intensity, and treatment schedule; molecular and cellular outcomes; behavioral outcomes; and any functional manipulation used to evaluate the proposed mechanism. The study-selection process is presented in Figure 2.

No anatomical restriction was applied during study selection. However, the eligible studies predominantly examined microglia in the spinal cord, particularly the dorsal horn; therefore, the spinal focus of this review reflects the available evidence rather than a prespecified anatomical criterion.

### 2.2. Risk-of-Bias and Mechanistic Evidence Appraisal

Two reviewers independently assessed study-level risk of bias using Systematic Review Centre for Laboratory Animal Experimentation (SYRCLE)’s risk-of-bias tool for animal intervention studies [70]. The tool evaluates ten domains: sequence generation, baseline characteristics, allocation concealment, random housing, blinding of caregivers and investigators, random outcome assessment, blinding of outcome assessors, incomplete outcome data, selective outcome reporting, and other sources of bias. Each domain was judged as low, unclear, or high risk of bias. Disagreements were resolved by discussion or consultation with a third reviewer. Each judgment was supported by a verbatim extract from the source article and a written justification. The complete assessment is provided in Table S2.

Mechanistic evidence was appraised separately according to the type of experimental support. Studies using loss-of-function approaches—including receptor antagonism, pharmacological inhibition, antibody or antiserum blockade, and gene knockdown—or gain-of-function approaches, such as agonist challenge, pathway activation, or overexpression, were classified as providing functional evidence. Studies reporting only changes in protein or mRNA expression, immunoreactivity, or phenotype-associated markers without direct pathway manipulation were classified as associative evidence. The number of contributing studies and consistency of behavioral findings were considered supportive context rather than primary indicators of causality.

Formal pathway-level certainty ratings using Grading of Recommendations Assessment, Development and Evaluation (GRADE) or Office of Health Assessment and Translation (OHAT) approach were not assigned because the included studies evaluated heterogeneous mechanisms using different animal models, EA protocols, molecular endpoints, and validation strategies, and many pathways were represented by only one or a few studies. Pathway-specific certainty ratings could therefore have implied a level of comparability not supported by the evidence.

## 3. Results

### 3.1. Search Results and Study Characteristics

The database search identified 2227 records: 48 from PubMed, 2098 from CINAHL, 81 from Web of Science, and none from the Cochrane Library. After removal of duplicates, 2218 records remained. Title screening excluded 2099 records, leaving 119 abstracts for assessment. Of these, 83 were excluded because they were reviews, conference abstracts without accessible full text, or studies involving irrelevant populations or conditions. Thirty-six full-text articles were assessed for eligibility; ten were excluded because the full texts could not be obtained despite attempts to contact the authors. Twenty-six studies met the eligibility criteria and were included in the review (Figure 2).

All were preclinical animal studies; 24 used rats and two used C57BL/6 mice, while 25 included male animals exclusively. The models comprised chronic constriction injury (CCI), spinal nerve ligation (SNL), spared nerve injury (SNI), chemotherapy-induced peripheral neuropathy (CIPN), diabetic neuropathic pain (DNP), and radicular pain.

EA protocols varied considerably in acupoint selection, stimulation frequency and intensity, and treatment duration. Most studies examined microglial responses in the spinal dorsal horn and assessed mechanical or thermal hypersensitivity together with molecular markers. The investigated mechanisms were grouped into four categories: initiation and purinergic activation, innate immune and inflammasome signaling, downstream inflammatory amplification, and pro-resolution neuroimmune modulation. Study characteristics are summarized in Table 1.

### 3.2. Risk of Bias and Appraisal of Mechanistic Evidence

**Figure 3:**
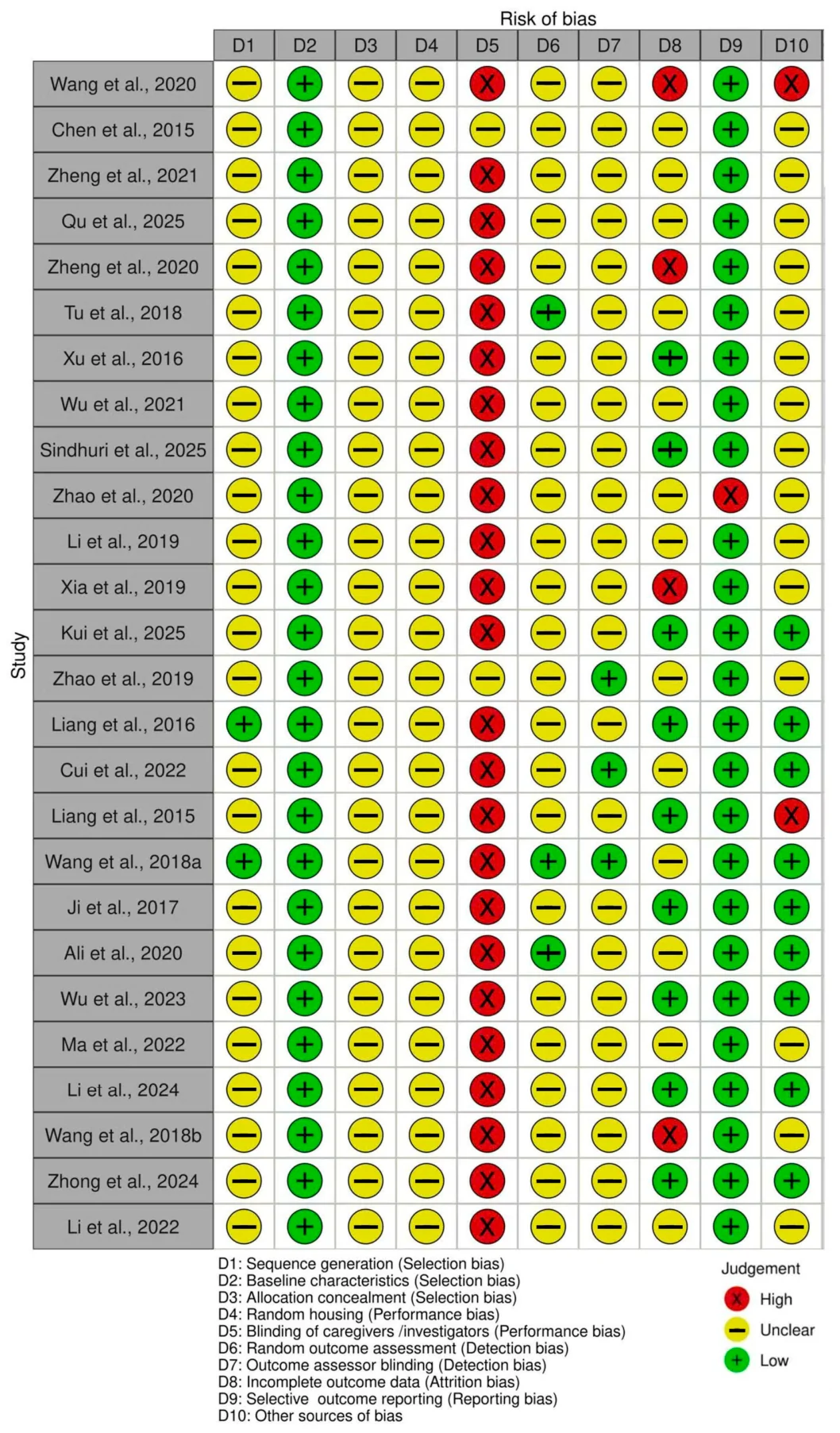
Study-level risk-of-bias judgments for the included animal studies. Each row represents an individual study, and each column represents one domain of SYRCLE’s risk-of-bias tool: D1, sequence generation; D2, baseline characteristics; D3, allocation concealment; D4, random housing; D5, blinding of caregivers and investigators; D6, random outcome assessment; D7, blinding of outcome assessors; D8, incomplete outcome data; D9, selective outcome reporting; and D10, other potential sources of bias. Green circles with a plus sign indicate low risk of bias, yellow circles with a minus sign indicate unclear risk of bias, and red circles with a multiplication sign indicate high risk of bias. Studies are identified in the figure by first author and year, and correspond, in the order shown from top to bottom, to Wang et al., 2020 [71]; Chen et al., 2015 [72]; Zheng et al., 2021 [73]; Qu et al., 2025 [74]; Zheng et al., 2020 [75]; Tu et al., 2018 [76]; Xu et al., 2016 [77]; Wu et al., 2021 [78]; Sindhuri et al., 2025 [79]; Zhao et al., 2020 [80]; Li et al., 2019 [81]; Xia et al., 2019 [82]; Kui et al., 2025 [83]; Zhao et al., 2019 [84]; Liang et al., 2016 [85]; Cui et al., 2022 [86]; Liang et al., 2015 [68]; Wang et al., 2018a [69]; Ji et al., 2017 [87]; Ali et al., 2020 [88]; Wu et al., 2023 [89]; Ma et al., 2022 [90]; Li et al., 2024 [91]; Wang et al., 2018b [92]; Zhong et al., 2024 [93]; and Li et al., 2022 [94].

**Figure 4:**
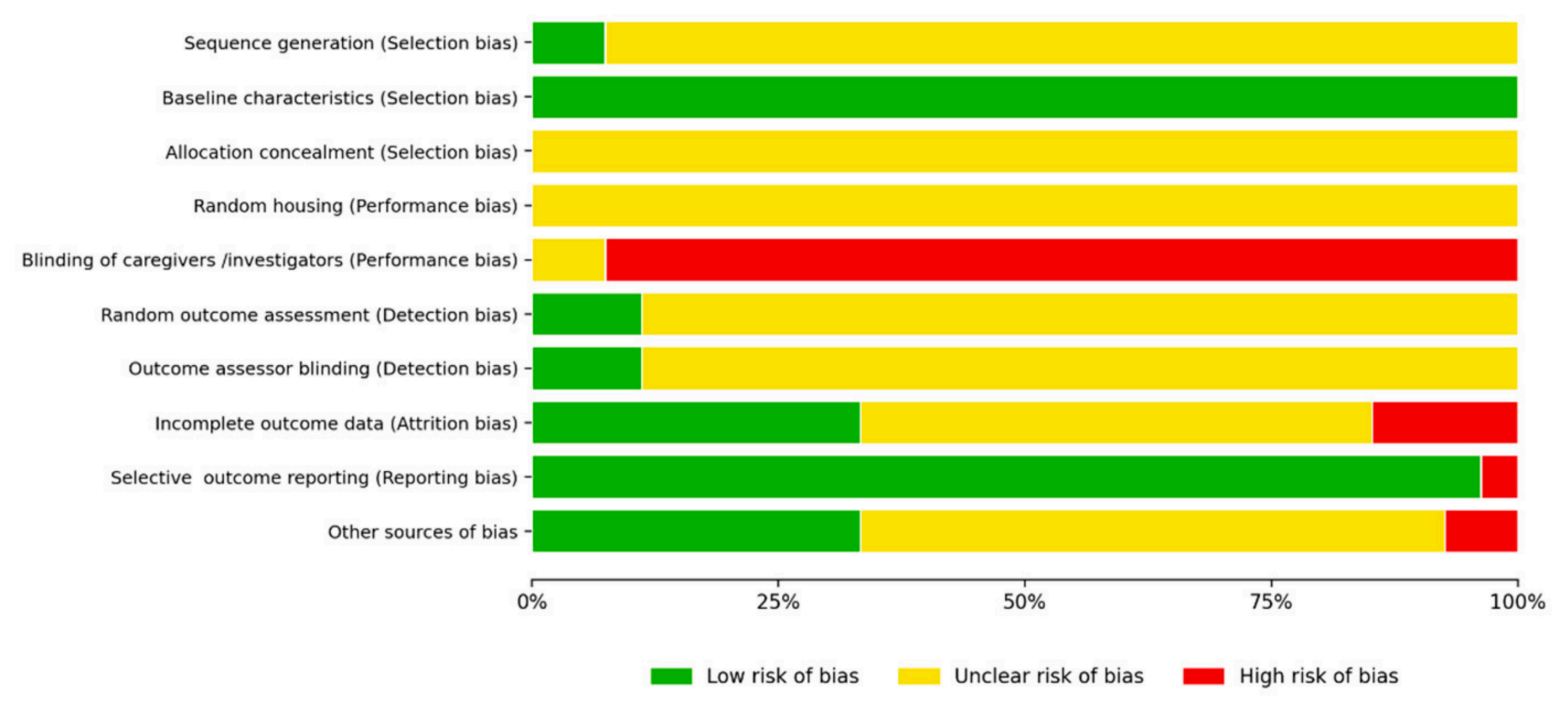
Summary of risk-of-bias judgments across the included animal studies. The stacked horizontal bars show the percentage of studies judged to have low, unclear, or high risk of bias in each domain of SYRCLE’s risk-of-bias tool. The assessed domains were sequence generation, baseline characteristics, allocation concealment, random housing, blinding of caregivers and investigators, random outcome assessment, blinding of outcome assessors, incomplete outcome data, selective outcome reporting, and other potential sources of bias. Green indicates low risk of bias, yellow indicates unclear risk of bias, and red indicates high risk of bias.

SYRCLE assessment identified important reporting and design limitations. Blinding of caregivers and investigators was judged to be at high risk in 92% of studies, allocation concealment and random housing were unclear in all studies, and four studies excluded animals using outcome-dependent criteria without complete reporting. Detailed judgments are presented in Figure 3 and Figure 4, and Table S2.

The methodological quality of these 26 studies was limited in several respects, and this constrains the strength of the mechanistic inferences that follow. Baseline characteristics were at low risk of bias in all 26 studies (100%), and 25 of 26 (96%) were free of selective outcome reporting. By contrast, blinding of caregivers and investigators was at high risk in 24 of 26 studies (92%). This is in part inherent to the intervention, since active electroacupuncture involves visible needle insertion and electrical stimulation and cannot be concealed from the operator; few studies, however, described separating the unblinded interventionist from personnel responsible for animal care or outcome assessment, which would have been feasible. Allocation concealment and random housing were unclear in all 26 studies (100%); sequence generation was unclear in 24 of 26 (92%); and random outcome assessment and blinding of outcome assessors were each unclear in 23 of 26 (88%), in most instances because randomisation or blinding was asserted without any description of the method used. Incomplete outcome data were at high risk in 4 studies (15%), principally because animals that failed to develop allodynia were excluded after group allocation without reporting the number, group of origin, or timing of those exclusions; because the exclusion criterion is itself the principal outcome, such attrition may bias treatment comparisons. Other sources of bias were judged high in 2 studies (8%), reflecting unit-of-analysis problems in which repeated or clustered observations were analysed as though independent. The synthesis below should therefore be read as a map of convergent mechanistic signals rather than as a set of effect estimates of established magnitude (Figure 4).

Mechanistic support varied across pathways. Functional evidence was available for P2X4R, P2X7R, NLRP3, PI3K/AKT, p38 MAPK, AMPK, IL-10/β-endorphin, PD-L1, GRK2, miR-124, α7nAChR, GLP-1R, and GABAᴀ receptor signaling [74,75,77,83,84,85,86,88,89,90,91,92,93,94]. In contrast, IRF8, BDNF–TrkB, TLR4/MyD88/NF-κB, COX-2, and time-dependent glial activation were supported mainly by treatment-associated expression changes [68,69,71,76,80,81,82,87]. The type of mechanistic validation and principal risk-of-bias concerns are summarized in Table 2.

### 3.3. Narrative Synthesis

**Figure 5:**
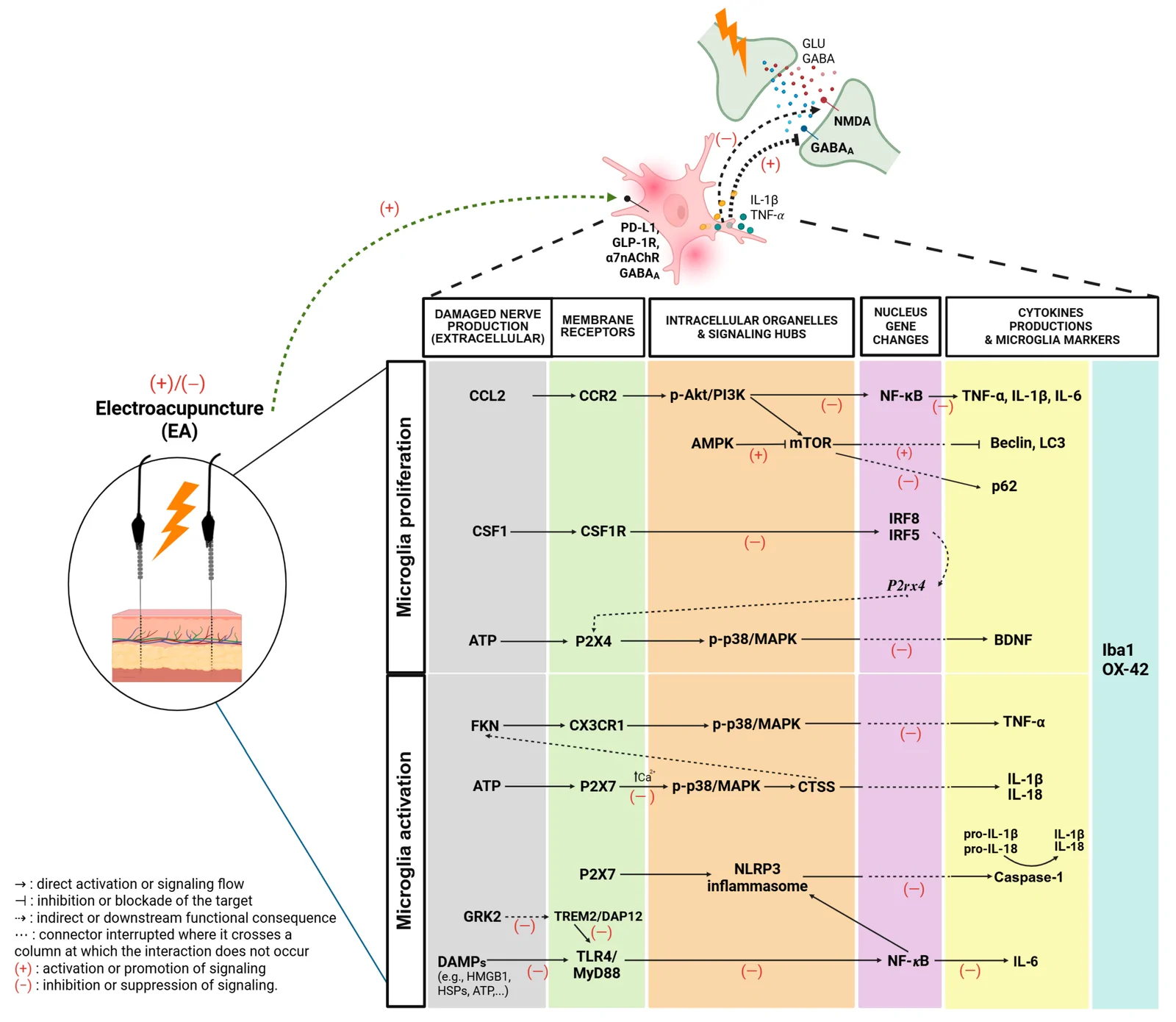
Proposed microglia-centered mechanisms of electroacupuncture in neuropathic pain. Peripheral nerve injury activates spinal microglia and associated purinergic, innate immune, inflammasome, and kinase-dependent inflammatory pathways, thereby promoting neuron–glia sensitization. EA, applied at an acupoints may attenuate these pain-promoting processes while enhancing pro-resolution mechanisms involving IL-10/β-endorphin, PD-L1/PD-1, GRK2/miR-124/TREM2/DAP12, α7nAChR, GLP-1R, and GABAergic signaling. At the synapse, EA attenuates microglial facilitation of NMDA-mediated excitation by glutamate (GLU) and is associated with increased GABAᴀ receptor subunit expression. Together, these effects may shift microglial activity toward a less inflammatory and more pro-resolution profile. Line styles and symbols are defined in the key; arrow type denotes the underlying signaling relationship in neuropathic pain, whereas (+) and (−) denote the direction of EA-associated modulation at that node. Both electrodes are drawn identically, and the color does not denote polarity or current direction. Vertical panels correspond to the levels named in the column headers, with the microglial markers Iba1 and OX-42 at the right; horizontal bands separate microglial proliferation (upper) from activation (lower). Connectors are routed for clarity, and colors group items visually without quantitative meaning. Gene names are italicized (*P2rx4*); all other labels denote proteins or small molecules. Akt, protein kinase B; AMPK, AMP-activated protein kinase; ATP, adenosine triphosphate; BDNF, brain-derived neurotrophic factor; Ca^2+^, calcium ion; caspase-1, cysteine-aspartic protease 1; CCL, C-C motif chemokine ligand; CCR, C-C motif chemokine receptor; CNS, central nervous system; COX-2, cyclooxygenase-2; CSF1, colony-stimulating factor 1; CSF1R, colony-stimulating factor 1 receptor; CTSS, cathepsin S; CX3CR1, CX3C chemokine receptor 1; DAMPs, damage-associated molecular patterns; DAP12, DNAX-activating protein of 12 kDa; FKN, fractalkine; GABA, γ-aminobutyric acid; GABAᴀ, γ-aminobutyric acid type A receptor; GLU, glutamate; GRK2, G protein-coupled receptor kinase 2; HMGB1, high-mobility group box 1; HSPs, heat-shock proteins; Iba1, ionized calcium-binding adapter molecule 1; IL, interleukin; iNOS, inducible nitric oxide synthase; IRF, interferon regulatory factor; LC3, microtubule-associated protein 1 light chain 3; MAPK, mitogen-activated protein kinase; MMP, matrix metalloproteinase; mTOR, mammalian target of rapamycin; MyD88, myeloid differentiation primary response 88; NF-κB, nuclear factor kappa B; NLRP3, NLR family pyrin domain containing 3; NMDA, N-methyl-D-aspartate receptor; NO, nitric oxide; OX-42, CD11b/c microglial marker; p-, phosphorylated; p62, sequestosome-1; *P2rx4*, purinergic receptor P2X4 gene; P2X4, P2X7 and P2Y12, purinergic receptors; PI3K, phosphoinositide 3-kinase; ROS, reactive oxygen species; TLR4, Toll-like receptor 4; TNF-α, tumor necrosis factor-α; TREM2, triggering receptor expressed on myeloid cells 2.

Twenty-six studies investigating EA effects on microglia-associated signaling pathways in neuropathic pain models were included. Despite substantial heterogeneity in animal models, acupoint combinations, stimulation parameters, and treatment duration, the studies included in this review revealed several convergent mechanistic patterns. Overall, EA was associated with reduced spinal microglial activation, suppression of pro-inflammatory mediators, attenuation of intracellular inflammatory signaling, and, in some studies, enhancement of pro-resolution neuroimmune pathways. This four-category framework was chosen to mirror the biological progression of microglia-associated signaling in neuropathic pain, from early activation and danger sensing to inflammatory amplification and resolution. Based on the dominant mechanistic entry points identified across the reviewed studies, the evidence can be interpreted within four major categories: initiation and purinergic microglial activation; innate immune sensing and inflammasome signaling; downstream inflammatory amplification and neuron-glia sensitization pathways; and pro-resolution and neuroimmune modulatory pathways (Figure 5). Throughout the sections that follow, a deliberate distinction is drawn between pathways in which the proposed target was functionally validated, by receptor antagonist, pharmacological inhibitor, antibody or antiserum blockade, gene knockdown, or overexpression, and pathways supported only by treatment-associated changes in marker expression. Pathways evaluated through loss- or gain-of-function manipulation were considered to have stronger evidence of functional involvement, whereas pathways supported only by treatment-associated expression changes were considered associative. Table 2 classifies every pathway discussed below on this basis.

#### 3.3.1. EA Regulates Initiation and Purinergic Microglial Activation

A recurring finding across multiple NP models is that EA attenuates early microglial activation and purinergic sensitization. Representative studies in Spared Nerve Injury (SNI), Chronic Constriction Injury (CCI), Spinal Nerve Ligation (SNL), and Diabetic Neuropathic Pain (DNP) models showed that EA decreased the expression of IRF8 [71], P2X4R [72,73,74,75], P2X7R [77,78], CD11b, OX-42, and Iba1, together with improvements in pain-related behaviors. These molecules are closely linked to the initiation of microglial activation after neural injury and contribute to the establishment of a pro-nociceptive spinal microenvironment. In particular, IRF8 is an important transcriptional regulator of reactive microglial phenotypes [21], whereas purinergic receptors such as P2X4R and P2X7R are key mediators of ATP-dependent microglial activation and neuron-microglia communication [46]. Thus, the repeated suppression of these pathways suggests that EA acts, at least in part, by interfering with the early molecular events that drive microglial priming and pain sensitization.

Among the purinergic pathways, the P2X4R axis was one of the most consistently reported. In CCI, SNL, and DNP models, EA reduced P2X4R expression together with markers of microglial activation [72], and in some studies this was accompanied by decreases in BDNF-related signaling [75] or excitatory synaptic activity, including reductions in spontaneous excitatory postsynaptic currents (sEPSCs) [73]. Because microglial P2X4R activation is known to promote BDNF release and facilitate central sensitization [25], these findings support the interpretation that EA suppresses a key receptor-level mechanism linking activated microglia to neuronal hyperexcitability. Importantly, some studies extended this observation beyond receptor expression alone. For example, EA-mediated suppression of P2X7R signaling was accompanied by reduced downstream p38 MAPK activation [77,78], while P2X4R-related studies also reported reductions in BDNF-associated sensitization [75,76], indicating that EA may influence not only upstream purinergic receptors but also their intracellular and functional consequences. One of these studies additionally reported increased GABA type A receptor subunit gamma 2 (GABAᴀγ2) expression together with failure to induce spinal long-term potentiation [75], a finding revisited in Section GABAergic Signaling because it links the purinergic axis to GABAergic disinhibition.

Functional evidence was uneven across this category. Involvement of the P2X4R axis was tested pharmacologically with the selective antagonist 5-BDBD [74,75], and P2X7R involvement was supported by A-438079 blockade together with experiments in cultured microglia [77]. Two further studies used gain-of-function challenges, with IFN-γ [72] and BzATP [78], which establish that these pathways are sufficient to drive microglial activation but do not by themselves demonstrate that EA acts through them. The IRF8/CX3CR1 [71] and BDNF–TrkB [76] findings rest on expression changes alone and are therefore best read as associations. Collectively, these findings indicate that EA attenuates the initiation and maintenance of microglia-associated nociceptive signaling by suppressing purinergic receptor pathways and their downstream effectors.

#### 3.3.2. EA Suppresses Innate Immune Sensing and Inflammasome Signaling

A second major mechanistic theme is the suppression of innate immune sensing pathways that enable microglia to detect injury-related danger signals and propagate neuroinflammation. Across several studies, EA reduced the expression of CCL2/CCR2, [79], HMGB1/TLR4/MyD88/NF-κB-related signaling [80,81,82], and NLRP3 inflammasome-associated molecules [83]. These pathways participate in chemokine recruitment, recognition of DAMPs, transcriptional activation of inflammatory mediators, and pyroptosis-related amplification of inflammation. Their repeated modulation by EA suggests that EA may reduce microglial responsiveness to injury-derived inflammatory stimuli and thereby restrain the transition from local damage signaling to persistent spinal neuroinflammation.

Among these pathways, TLR4/MyD88-related signaling appears particularly relevant. In paclitaxel-induced NP and nerve injury models, EA reduced TLR4 and MyD88 expression together with OX-42/CD11b-positive microglial activation and other inflammatory readouts, indicating attenuation of an important upstream innate immune signaling cascade [81,82]. Because TLR4/MyD88 signaling activates downstream inflammatory transcriptional programs and contributes to cytokine production, microgliosis, and pain hypersensitivity [40], its suppression provides a plausible mechanistic explanation for the anti-neuroinflammatory effects of EA. Similarly, the reduction in chemokine axes such as CCR2 supports the interpretation that EA weakens the recruitment and reinforcement of inflammatory signaling networks that sustain NP [79].

The NLRP3 inflammasome study provides especially valuable mechanistic support because it moved beyond descriptive marker changes. In CCI rats, EA reduced NLRP3, cleaved caspase-1, N-terminal gasdermin D (N-GSDMD), IL-18, and IL-1β, indicating suppression of inflammasome activation and microglial pyroptosis [83]. Importantly, pharmacological inhibition of NLRP3 with MCC950 further supported the involvement of inflammasome signaling, suggesting that suppression of NLRP3-mediated pyroptosis is functionally related to the analgesic effect of EA [83].

Functional support in this category is concentrated in the inflammasome arm, where pharmacological inhibition with MCC950 provided additional support for the involvement of NLRP3 signaling in the observed anti-pyroptotic and analgesic effects [83], and in the CCL2/CCR2 axis, which was probed with intrathecal recombinant CCL2 [79]. Notably, TLR4 itself was never directly manipulated: the only pharmacological intervention among the TLR4-related studies targeted TRPV1 [81], while the TLR4/NF-κB [80] and HMGB1/TLR4–MyD88 [82] findings are based on expression changes alone. The TLR4/MyD88 mechanism should therefore be regarded as consistently associated with EA treatment rather than as causally established. Taken together, the findings in this category suggest that EA suppresses microglia-associated neuroinflammation not only by attenuating receptor-level activation, but also by limiting injury sensing, inflammatory transcription, and inflammasome-dependent escalation of the inflammatory response.

#### 3.3.3. EA Attenuates Downstream Inflammatory Amplification and Neuron-Glia Sensitization

In addition to its effects on early activation pathways, EA also appears to inhibit downstream intracellular signaling cascades that amplify inflammation and promote neuron-glia sensitization. Across the reviewed studies, EA modulated PI3K/AKT [84], p38 MAPK signaling [85], AMP-activated protein kinase (AMPK)/mTOR-associated autophagy pathways [86], COX-2 [87], and broader patterns of spinal microglial and astrocytic activation [68,69]. These pathways are not all exclusively microglia-specific, but together they represent a broader inflammatory amplification network that contributes to the maintenance of NP and central sensitization. The convergence of these downstream findings suggests that EA acts beyond initial receptor suppression and may interrupt the intracellular signaling machinery required to sustain chronic neuroinflammatory pain states.

Among these downstream pathways, p38 MAPK is one of the most recurrent effectors. Several studies reported that EA reduced p-p38 expression together with OX-42/Iba1-positive microglial activation, pro-inflammatory cytokine production, and pain-related hypersensitivity (77, 78, 85). This is mechanistically important because p38 MAPK is a well-established mediator of inflammatory signal transduction in activated microglia and is closely linked to cytokine release and central sensitization. In a similar way, suppression of PI3K/AKT signaling after EA was associated with reduced Iba1, IL-1β, and TNF-α, further supporting the notion that EA attenuates intracellular programs involved in neuroinflammatory amplification [84]. Although the AMPK/mTOR-autophagy findings may represent a somewhat different signaling context, they are also consistent with the interpretation that EA influences cell-stress and inflammatory regulatory pathways relevant to glial activation [86].

Additional support for EA-mediated suppression of downstream inflammatory amplification comes from a study examining COX-2 [87]. COX-2 is a major inducible enzyme responsible for prostanoid generation within the spinal inflammatory milieu. In the study summarized, EA reduced spinal COX-2, OX-42, and GFAP, together with improvements in pain-related behaviors. These findings suggest that EA suppresses not only intracellular inflammatory cascades, but also downstream enzymatic systems that sustain the neuroimmune microenvironment required for persistent NP. Moreover, studies describing suppression of early microglial activation together with later astrocytic activation imply that EA may influence the temporal evolution of glial responses during NP progression [68,69].

Each of the three intracellular kinase pathways in this category was probed pharmacologically—LY294002 for PI3K [84], SB203580 for p38 MAPK [85], and compound C for AMPK [86]—placing them among the better-supported downstream mechanisms. The COX-2 [87] and microglial-to-astrocytic time-course findings [68,69] rest on expression changes alone and are correspondingly weaker as causal claims. Taken together, the evidence in this subsection supports a model in which EA suppresses not only upstream activation signals, but also the downstream intracellular and intercellular pathways that amplify neuroinflammation and reinforce persistent pain.

#### 3.3.4. EA Promotes Pro-Resolution and Neuroimmune Modulatory Pathways

Beyond suppressing pro-inflammatory signaling, an important subset of studies suggests that EA also activates pathways that actively favor inflammatory resolution, endogenous analgesia, and restoration of spinal neuroimmune homeostasis. These mechanisms include the spinal IL-10/β-endorphin pathway, programmed death-ligand 1 (PD-L1) -associated microglial phenotype switching, GRK2- and miR-124-dependent regulation of TREM2/DAP12 signaling, α7 nicotinic acetylcholine receptor (α7nAChR) signaling, glucagon-like peptide-1 (GLP-1)/GLP-1 receptor (GLP-1R) signaling, and GABAergic inhibitory mechanisms (Table 1 and Table 2). Importantly, most of these targets were reported in relation to spinal microglia, whereas the GABAergic pathway appears to act primarily at the level of spinal dorsal horn neurons. Thus, unlike earlier sections that focus mainly on inhibition of inflammatory activation, this category highlights the possibility that EA not only suppresses pathogenic signaling but also recruits intrinsic recovery-associated programs.

##### IL-10/β-Endorphin Pathway

The IL-10/β-endorphin pathway represents a classic pro-resolution mechanism linking immune regulation to endogenous analgesia. IL-10 is a potent anti-inflammatory cytokine that suppresses the production of pro-inflammatory mediators and limits glial activation, whereas β-endorphin is an endogenous opioid peptide that reduces nociceptive transmission through μ-opioid receptor-dependent mechanisms [57,88]. In NP, enhancement of this pathway is particularly relevant because it can simultaneously dampen neuroinflammation and strengthen endogenous pain inhibition. In the included study, EA increased spinal IL-10 and β-endorphin expression together with upregulation of opioid peptide-related genes and improvement in pain behavior [88]. Mechanistically, this effect was not merely associative: blockade with an IL-10 antibody, β-endorphin antiserum, or the selective μ-opioid receptor antagonist CTAP abolished the analgesic effect of EA. These findings support the interpretation that EA promotes a functionally important pro-resolution pathway involving spinal microglial IL-10/β-endorphin signaling rather than simply reducing inflammation indirectly.

##### PD-L1/PD-1 Signaling and Microglial Phenotype Transition

PD-L1/PD-1 signaling is increasingly recognized as an immune checkpoint pathway with protective functions in NP. In the spinal cord, activation of this pathway has been associated with suppression of pro-inflammatory microglial activity, reduced MAPK and NF-κB signaling, and promotion of a shift away from the M1-like phenotype toward a more pro-resolving M2-like state. This is biologically important because persistent M1-like polarization sustains cytokine release and neuronal sensitization, whereas M2-associated polarization is generally linked to inflammation resolution and tissue repair. In the relevant study [89], EA increased PD-L1 and PD-1 expression, reduced Iba1 and pro-inflammatory cytokines, and promoted expression of M2-associated markers such as arginase-1 (Arg-1) and CD206 while reducing M1-associated markers including CD16 and iNOS [89]. The mechanistic role of this pathway was strengthened by knockdown evidence showing that disruption of PD-L1 reversed the beneficial effects of EA. Therefore, EA appears to promote analgesia at least in part by engaging an immune checkpoint mechanism that restrains spinal microglial inflammatory activation and supports phenotype transition toward a more reparative state.

##### GRK2/TREM2/DAP12 Signaling

G protein-coupled receptor kinase 2 (GRK2) is not a receptor but a regulatory kinase that plays an important role in controlling inflammatory signaling and neuron-glia communication. In NP conditions, reduced GRK2 signaling has been associated with enhanced spinal neuroinflammation and persistent pain hypersensitivity [95,96]. The study of Ma et al. suggests that GRK2 is particularly relevant because it functionally intersects with the TREM2/DAP12 axis, a signaling complex involved in regulating microglial reactivity [90]. In the CIPN model, EA increased GRK2 expression while reducing TREM2, DAP12, IL-1β, IL-6, iNOS, and the M1-associated marker CD16, together with behavioral improvement [90]. Importantly, neuron-specific GRK2 knockdown abolished the protective effect of EA, indicating that this pathway is functionally required rather than merely correlated with treatment response. Although the upstream manipulation in this model involved neuronal GRK2, the downstream consequences included attenuation of spinal microglial inflammatory activation, suggesting that EA may regulate microglial TREM2/DAP12-dependent reactivity through a neuron-to-microglia modulatory mechanism.

##### miR-124/TREM2/DAP12 Regulation

miR-124 is a well-known anti-inflammatory microRNA that contributes to maintenance of microglial quiescence and suppression of neuroinflammatory signaling. In NP, downregulation of miR-124 is associated with enhanced microglial activation and increased expression of pro-inflammatory pathways [91]. In the included study, EA increased spinal neuronal miR-124 expression while reducing TREM2, DAP12, iNOS, and M1-associated CD16/32, and simultaneously increasing IL-10 with reductions in TNF-α and IL-1β [91]. Functionally, downregulation of neuronal miR-124 weakened or prevented the beneficial effects of EA, supporting a causal role for this regulatory mechanism. Similar to the GRK2 study, these findings suggest that EA does not act only by directly suppressing inflammatory microglia but may also engage neuron-derived regulatory programs that secondarily restrain microglial TREM2/DAP12-associated activation. This pathway is particularly important because it provides a post-transcriptional mechanism through which EA may shift the spinal neuroimmune environment toward resolution.

##### α7 Nicotinic Acetylcholine Receptor (α7nAChR)

The α7nAChR is a cholinergic anti-inflammatory receptor that has been implicated in suppression of cytokine production and glial inflammatory activation. In the spinal cord, α7nAChR signaling is thought to restrain neuroinflammation and thereby reduce nociceptive sensitization [97]. In an SNI rat model, 2 Hz EA increased spinal α7nAChR expression, reduced IL-1β production and CD11b-positive microglial activation and improved mechanical hypersensitivity [92]. The functional involvement of α7nAChR was supported by intrathecal administration of α-bungarotoxin, a selective α7nAChR antagonist, which blocked the analgesic effect of EA and prevented EA-induced changes in α7nAChR and IL-1β expression. These findings suggest that EA may alleviate neuropathic pain through an α7nAChR-associated anti-inflammatory pathway that suppresses spinal microglial activation and IL-1β-mediated neuroinflammation.

##### GLP-1/GLP-1R Signaling

GLP-1 and its receptor GLP-1R are best known for metabolic functions, but increasing evidence suggests that they also exert anti-inflammatory and neuroprotective effects in the CNS. In NP, GLP-1R activation has been associated with reduced glial activation and attenuation of spinal hypersensitivity [98]. In the included study, EA increased GLP-1 and GLP-1R expression while reducing Iba1-positive microglial activation and improving pain-related behaviors [93]. Pharmacological blockade of GLP-1R reversed the beneficial effect of EA, indicating functional involvement of this pathway. Because the study linked GLP-1/GLP-1R signaling to reduced spinal glial activation, this mechanism is most appropriately interpreted as another example of EA promoting an anti-inflammatory microglial regulatory pathway rather than merely suppressing pain transmission downstream.

##### GABAergic Signaling

In contrast to the pathways above, the GABAergic mechanism appears to act primarily at the level of spinal dorsal horn neurons, rather than directly on microglia. GABA is the principal inhibitory neurotransmitter in the spinal cord, and impairment of GABAergic signaling is a major contributor to central sensitization in NP. Restoration of GABA receptor function is therefore highly relevant because it helps rebalance excitatory and inhibitory transmission within spinal circuits [99]. In the included study, EA increased expression of GABA type A (GABAᴀ) receptor subunits and improved pain behavior, while blockade of GABAᴀ receptors interfered with the analgesic effect [94]. This suggests that part of EA-induced analgesia depends on restoration of neuronal inhibitory control. Independent expression-level support comes from a study categorised above under purinergic signaling; in SNL rats, EA increased spinal GABAᴀγ2 while concurrently reducing P2X4R and BDNF, and spinal long-term potentiation was not induced [75]. This convergence is mechanistically coherent. As outlined in Section 1.2, microglial BDNF acting at TrkB downregulates KCC2 and collapses the neuronal chloride gradient, so that GABAergic transmission loses its hyperpolarizing effect. A reduction in BDNF accompanied by increased GABAᴀγ2 expression is therefore consistent with EA relieving microglia-driven disinhibition rather than acting on GABAᴀ receptors directly. Two points temper this interpretation: the causal manipulation in that study targeted P2X4R (5-BDBD) rather than GABAᴀ signaling, so it contributes expression-level rather than functional evidence, and KCC2 itself was not measured. Nonetheless, the two studies converge on increased GABAᴀγ2 expression in two different models, SNL [75] and CCI [94]. Accordingly, this pathway is best discussed as a neuron-centered inhibitory mechanism rather than a direct microglial receptor pathway, although the convergent findings above suggest that it may be functionally coupled to the microglial P2X4R/BDNF axis rather than independent of it.

## 4. Discussion

NP is increasingly understood as a disorder involving both abnormal neuronal activity and maladaptive neuroimmune interactions in the spinal cord, in which microglia contribute to the development and maintenance of central sensitization [6,9,10]. The principal finding of this review is that EA-associated analgesia converges on coordinated regulation of spinal neuroimmune signaling rather than on a single microglial target. Across different neuropathic pain models, EA was consistently associated with reduced microglial reactivity and inflammatory signaling, attenuation of neuron–glia sensitization, and enhancement of selected pro-resolution and inhibitory mechanisms. The four proposed mechanistic categories—initiation and purinergic microglial activation [71,72,73,74,75,76,77,78], innate immune and inflammasome signaling [79,80,81,82,83], downstream inflammatory amplification and neuron–glia sensitization [84,85,86,87], and pro-resolution neuroimmune modulation—provide a useful framework for organizing the evidence [88,89,90,91,92,93,94]. However, they should not be interpreted as a proven temporal cascade. Because most studies examined one pathway in isolation, the available evidence does not establish whether EA modulates these mechanisms sequentially, simultaneously, or through convergence on shared regulatory nodes. Figure 5 is therefore best viewed as an integrated network model rather than a linear signaling sequence.

Functional validation was unevenly distributed across the four categories. Pro-resolution mechanisms were more frequently examined using loss- or gain-of-function approaches, whereas several initiation and inflammatory-amplification pathways remained supported mainly by expression-level findings. Thus, the overall framework integrates pathways with different degrees of functional support.

Pain processing extends beyond the spinal cord to supraspinal networks that regulate its sensory, affective, and cognitive dimensions, while descending pathways from regions such as the periaqueductal gray and rostral ventromedial medulla modulate nociceptive transmission in the spinal dorsal horn. Reducing spinal microglial activation, inflammatory mediator release, and microglia-associated disinhibition would be expected to decrease the ascending nociceptive input reaching the thalamus, cortex, amygdala, and other pain-processing regions. Microglial activation has also been observed in the medial prefrontal cortex, amygdala, and hippocampus at delayed stages of neuropathic pain, although such responses are region- and time-dependent and are not consistently detected throughout the brain [100]. Several mechanisms identified in the included studies may interface with these broader pain-control systems. Most notably, EA-induced activation of a spinal microglial IL-10/β-endorphin pathway was functionally supported by IL-10 antibody, β-endorphin antiserum, and μ-opioid receptor blockade, thereby linking local neuroimmune modulation to endogenous opioid analgesia [88]. Other spinal mechanisms involving α7nAChR [92], GLP-1R [93], GABAergic signaling [94], and P2X4R–BDNF regulation [75] may similarly interact with descending modulation or reduce the ascending nociceptive drive reaching supraspinal regions [101,102,103,104,105,106]. However, all included studies investigated these mechanisms at the spinal level. None directly assessed brain microglia, supraspinal nuclei, or descending projections. Therefore, the included evidence supports spinal modulation of nociceptive signaling but does not establish that EA directly regulates supraspinal microglial activity.

Taken together, the findings support a multi-level framework in which EA attenuates spinal microglial inflammatory signaling while enhancing selected pro-resolution and inhibitory mechanisms. However, individual pathways differ substantially in functional validation and independent replication. Future studies should combine pathway-specific manipulation with longitudinal behavioral and molecular measurements to determine how these mechanisms interact and whether the observed microglial changes precede, accompany, or follow EA-induced analgesia.

## 5. Limitations

Several limitations qualify the conclusions of this review. The evidence base is methodologically constrained. As detailed above, blinding of caregivers and investigators was judged to be at high risk of bias in 92% of the included studies, allocation concealment and random housing were unclear in all studies, and four studies excluded animals according to outcome-dependent criteria without complete reporting (Figure 3 and Figure 4; Table S2). Randomisation and blinding were frequently stated without a description of the methods used; therefore, the true risk of bias may in some cases be lower than the “unclear” classification suggests. Nevertheless, incomplete reporting prevents reliable assessment of study conduct and limits confidence in individual findings, consistent with the rationale underlying SYRCLE and ARRIVE 2.0 [70,107].

Although study-level risk of bias was formally assessed using SYRCLE, no pathway-level certainty rating was assigned using GRADE or OHAT. GRADE has been adapted for preclinical intervention studies, but methodological challenges remain when evaluating heterogeneous animal evidence, while OHAT is principally structured around defined exposure–health-outcome evidence and applies project-specific approaches to mechanistic evidence [108,109]. The heterogeneity of the models, EA protocols, molecular endpoints, and validation strategies, together with the small number of studies supporting many pathways, limited meaningful body-of-evidence grading. Mechanistic conclusions should therefore be interpreted according to the type of experimental validation and consistency of findings rather than as formal certainty ratings.

Considerable methodological heterogeneity also restricted quantitative synthesis. Differences in pain models, acupoint selection, stimulation parameters—ranging from 2 Hz to alternating 2/100 Hz and from 0.5 to 3 mA—and treatment schedules extending from a single session up to 16 sessions precluded meta-analysis and prevented firm conclusions regarding parameter-specific effects. It remains unclear which parameters are necessary, which are merely sufficient, and how far a protocol can be modified before its effect is lost. Systematic dose-finding and parameter-mapping studies are needed to improve reproducibility and support translation.

The limited representation of sex and species further constrains generalisability. Twenty-five of the 26 studies used male animals exclusively, and only one included females. Experimental evidence indicates that spinal microglia are required for mechanical pain hypersensitivity in male but not female mice, with adaptive immune cells contributing more prominently in females; sex-dependent involvement of microglial P2X4R signalling has also been demonstrated in rats [110,111,112]. The microglia-centered framework proposed here may therefore not generalise to female animals, and its relevance to women remains uncertain. Species and strain diversity was similarly narrow: 24 studies used rats, predominantly Sprague–Dawley rats, two used C57BL/6 mice, and no large-animal mechanistic data were available.

The mechanistic scope of the evidence was also limited. Most studies interrogated a single pathway at a time; consequently, the manner in which the four mechanistic categories interact in vivo, and whether they represent parallel, sequential, or overlapping consequences of EA, cannot be determined from the present evidence. The search was restricted to English-language publications from January 2015 to November 2025. Although this provided a focused synthesis of contemporary evidence, it may have excluded relevant earlier EA studies and work published in other languages, particularly given the geographical distribution of acupuncture research. Publication bias toward positive findings cannot be excluded. The temporal dimension also remains undercharacterised: the optimal timing of intervention relative to nerve injury, the persistence of effects after treatment cessation, and the durability of microglial phenotypic changes have been addressed only partially.

For these reasons, the proposed model should be regarded as a framework for organising the current preclinical evidence and generating testable hypotheses rather than as an established account of how EA relieves neuropathic pain in patients. Clinical translation will require studies that pair behavioral or clinical endpoints with mechanistic readouts, such as cerebrospinal-fluid inflammatory markers or translocator protein positron emission tomography, to examine whether the neuroimmune mechanisms characterised in rodents are also operative in human neuropathic pain [113].

## 6. Conclusions

Overall, the available preclinical evidence supports a model in which EA attenuates neuropathic pain by modulating microglia-associated purinergic activation, innate immune sensing, inflammasome signaling, kinase-dependent inflammatory amplification, and pro-resolution neuroimmune pathways. Future studies should define parameter-specific effects, verify cell-specific mechanisms, and integrate standardized animal reporting with clinically relevant translational endpoints.

## Figures and Tables

**Table 1:** Characteristics of the 26 included electroacupuncture studies.

Study (Ref)	Model	Animal	EA Points	Freq	Intensity	Duration/ Schedule	Pathways + Readout	Microglia Measure	Behavioral Readout	Functional Evidence
Wang 2020 [71]	SNI	Rat SD, M	ST36 + SP6 (bil)	2 Hz	1–3 mA	30 min, q.o.d ×21 d	↓IRF8 ↓CX3CR1	- IF: L4-L6 spinal cord, ↓IRF8/CD11b, CX3CR1/CD11b	↑PWT	No (marker only)
Chen 2015 [72]	CCI; i.t. IFN-γ	Rat SD, M	GB30 (bil)	2 Hz	2 mA	30 min, daily ×14 d	↓P2X4R ↓IFN-γ	- IHC: L4-L5 spinal cord, ↓P2X4R/OX-42	↑PWT	IFN-γ challenge (gain-of-fn)
Zheng 2021 [73]	SNL	Rat SD, M	ST36 + BL60 (ipsi)	2/100 Hz	1–1.5 mA	30 min, daily ×7 d	↓P2X4R ↓sEPSC frequency	- IF: L4-L5 spinal cord, ↓P2X4R/Iba1	↑MWT, TWL	No (marker + ex vivo ephys)
Qu 2025 [74]	DNP (STZ)	Rat SD, M	ST36 + BL60	2 Hz	1 mA	30 min, daily ×7 d	↓P2X4R, ↓BDNF ↓TNF-α, ↓IL-1β	- IF: spinal cord, ↓CD11b, - WB: spinal cord, ↓Iba1	↑PWL	5-BDBD (P2X4R antag.) Minocycline (Microglia antag.)
Zheng 2020 [75]	SNL	Rat SD, M	ST36 + BL60	2/100 Hz	1.5 mA	30 min, daily ×7 d	↓P2X4R, ↓BDNF ↑GABAᴀγ2 LTP not induced	- IF+ WB+ RT-qPCR: L4-L6 spinal cord, ↓Iba1	↑MWT, TWL	5-BDBD (P2X4R antag.)
Tu 2018 [76]	CCI	Rat SD, M	ST36 + GB34 (ipsi)	2/100 Hz	1.5 mA	30 min, daily ×7 d	↓BDNF ↓TrkB	- IF: L4-L6 spinal cord, ↓BDNF/Iba1 - RT-qPCR: L4-L6 spinal cord, ↓Iba1	↑MWT, TWL	No (marker only)
Xu 2016 [77]	CCI; i.t. BzATP	Rat SD, M	GB30 (bil)	2 Hz	2 mA	30 min, daily ×14 d	↓P2X7R ↓IL-1β ↓IL-18	- IF: L4-L5 spinal cord, ↓P2X7R/OX-42	↑PWT, PWL	A-438079 (P2X7R antag.) + culture
Wu 2021 [78]	SNL; i.t. BzATP	Rat SD, M	ST36 + BL60	2 Hz	1.5 mA	30 min, daily ×7 d	↓P2X7R, p-p38 MAPK ↓BDNF, IL-1β, IL-6, TNF-α ↑IL-10	- WB+ IF: L4-L6 spinal cord, ↓Iba1	↑MWT, TWL	BzATP challenge (gain-of-fn)
Sindhuri 2025 [79]	SNL	Rat SD, M	ST36 + GB34 (contra)	2 Hz	1 mA	30 min, daily ×5 d	↓CCL2, CCR2 ↓IL-1β, TNF-α	- IF: L4-L6 spinal cord, ↓Iba1	↑PWT, PWL	i.t. recombinant CCL2 (challenge)
Zhao 2020 [80]	CIPN (PTX)	Rat SD, M	ST3 (bil)	10 Hz	1 mA	30 min, q.o.d ×14 d	↓TLR4 ↓NF-κB, p65 ↓IL-1β ↓TNF-α ↓GFAP	- WB+ ELISA: lumbar spinal cord, ↓TMEM119	↑MWL	No (marker only)
Li 2019 [81]	CIPN (PTX)	Rat SD, M	ST36 + BL60 (bil)	2 Hz	0.5–1.5 mA	30 min, daily ×7 d	↓TLR4, MyD88, TRPV1 ↓capsaicin-induced Ca2+ response, GFAP	- IF: L4–6 DRGs and spinal cord,↓OX-42	↑PWT, PWL	AMG9810/capsaicin (TRPV1)
Xia 2019 [82]	SNI	Rat SD, M	ST36 + SP6 (bil)	2 Hz	1–3 mA	30 min, q.o.d ×21 d	↓HMGB1 ↓TLR4, ↓MyD88 ↓NF-κB p65; p-NF-κB p65	- IF: L4-L6 spinal cord, ↓TLR4/CD11b, ↓MyD88/CD11b	↑PWT; gait	No (marker only)
Kui 2025 [83]	CCI	Rat SD, M	GV20 + ST36 (ipsi)	2 Hz	1 mA	20 min, daily ×14 d	↓NLRP3, ↓N-GSDMD, ↓Cleaved Caspase-1 ↓IL-18 ↓IL-1β ↓TNF-α	- IF: L4-L6 spinal cord, ↓NLRP3/Iba1, Caspase-1/Iba1, N-GSDMD/Iba1	↑MWT, PWL	MCC950 (NLRP3 inhib.)
Zhao 2019 [84]	CCI	Rat SD, M	ST36 + GB34	2 Hz	1 mA	30 min, daily ×14 d	↓p-PI3K/p-AKT ↓TNF-α ↓IL-6	WB: L4-L5 spinal cord, ↓Iba1	↑PWT, TWL	LY294002 (PI3K inhib.)
Liang 2016 [85]	SNL	Rat SD, M	ST36 + BL60 (bil)	2/100 Hz	1–2 mA	30 min, daily ×3 d	↓p-p38 MAPK	Double IF: L4-L6 SDH, ↓p-p38 MAPK/OX-42	↑PWT	SB203580 (p38 inhib.)
Cui 2022 [86]	SNI	Rat SD, M	ST36 + GB30 (ipsi)	2 Hz	1–3 mA	30 min, daily ×7 d	↑pAMPK/AMPK, ↓p-mTOR/mTOR; ↓p62 ↑Beclin-1 ↑LC3-II/LC3-I	TEM: ipsilateral lumbar spine L4-L5 ↑ autophagosomes	↑PWT	Compound C (AMPK inhib.)
Liang 2015 [68]	SNL	Rat SD, M	ST36 + BL60 (bil)	2/100 Hz	1–2 mA	30 min, daily to d14	↓GFAP (days 3–14 post-ligation)	IF: L4-L6 SDH, ↓OX-42 (day 3 post-ligation)	↑PWT	No (marker only)
Wang 2018 [69]	CCI	Rat Wistar, M	ST36 + GB34 (bil)	2/15 Hz	1 mA	30 min, daily ×14 d	↓GFAP (1–2 weeks after EA)	IF: L4-L5 SDH, ↓Iba1 (2 days after EA)	↑Mech/thermal PT	No (marker only)
Ji 2017 [87]	Nucleus pulposus (L5)	Rat SD, M	EX-B2 + BL25/ 40/60	2/100 Hz	1–3 mA	20 min, daily ×7 d	↓COX-2	- IF: L4-L6 spinal cord, ↓COX-2/OX-42	↑TWL	No (marker; EA-point variants)
Ali 2020 [88]	SNL	Rat Wistar, M + F	ST36 + SP6 (ipsi)	2 Hz	2–3 mA	20 min, single	↑IL-10 ↑β-endorphin ↑POMC, PNOC, PENK, no change in PDYN	- IF: spinal dorsal horn/L3–L5, ↑IL-10/Iba1, ↑β-endorphin/Iba1	↑MWT	IL-10 Ab, β-EP antiserum, CTAP
Wu 2023 [89]	SNL	Rat SD, M	ST36 + BL60 (ipsi)	2/100 Hz	1.5 mA	30 min, daily ×7 d	↑PD-L1, PD-1 ↓p-p38, p-ERK1/2, p-JNK, ↓p-NF-κB ↑IL-10 ↓IL-1β ↓IL-6 ↓TNF-α	- WB +IF: L4-L6 spinal cord, ↓Iba1; ↑Arg-1, ↑CD206; ↓CD16, ↓iNOS	↑MWT, TWL	PD-L1 siRNA (knockdown) + BV2
Ma 2022 [90]	CIPN (cisplatin)	Mouse C57BL/6, M	ST36 + BL60 (bil)	2/100 Hz	1–2 mA	30 min, daily ×16 d	↑ GRK2, ↓IENF loss ↓TREM2, DAP12 ↓IL-1β, IL-6, iNOS	- RT-qPCR: L4-L6 spinal cord, ↓CD16	↑PWT; ↓adhesive	Neuronal GRK2 OE/KD (AAV)
Li 2024 [91]	CIPN (cisplatin)	Mouse C57BL/6, M	ST36 + BL60 (bil)	2/100 Hz	1–2 mA	30 min, daily ×7 d	↑miR-124, ↓IENF loss ↓TREM2, DAP12, iNOS ↑IL-10, ↓TNF-α ↓IL-1β	- qPCR: spinal dorsal horn, ↓CD16/32, ns CD206 ↓iNOS	↑PWT; ↓adhesive	Neuronal miR-124 OE/KD (AAV)
Wang 2018 [92]	SNI	Rat SD, M	ST36 + SP6 (bil)	2 Hz	1–3 mA	30 min, q.o.d ×10 d	↑α7nAChR ↓IL-1β	- IF: L4-L6 spinal dorsal horn, ↑α7nAChR ↓CD11b	↑PWT	α-bungarotoxin (α7nAChR antag.)
Zhong 2024 [93]	SNI	Rat SD, M	ST36 + SP6 (bil)	2 Hz	1 mA	30 min, daily ×14 d	↑GLP-1/GLP-1R ↓GFAP	- IF +WB: L4-L6 spinal dorsal horn, ↓Iba1	↑PWT, TWL	Exendin-4/exendin-(9–39)
Li 2022 [94]	CCI	Rat SD, M	ST36 + GB34	2/100 Hz	1.5 mA	30 min, daily ×7 d	↑GABAᴀα2, ↑GABAᴀα3, ↑GABAᴀγ2	- WB +IF: L4-L6 spinal cord, ↓Iba1	↑MWT, TWL	Bicuculline (GABAᴀ antag.)

Arrows indicate the direction of change reported in the electroacupuncture group relative to the untreated model group: ↓ denotes a decrease and ↑ an increase in the expression, phosphorylation, or immunoreactivity of the indicated molecule or marker. Where an arrow refers to a model-induced rather than a treatment-induced change, this is stated explicitly within the cell. In the behavioral-outcome column, the arrow denotes the direction of the measured variable rather than the severity of pain: ↑PWT, ↑MWT, ↑PWL, ↑TWL and ↑MWL (increased withdrawal thresholds or latencies), and ↓adhesive removal time, all indicate attenuation of pain-related hypersensitivity or neuropathy. 5-BDBD, 5-(3-bromophenyl)-1,3-dihydro-2H-benzofuro [3,2-e]-1,4-diazepin-2-one; AAV, adeno-associated virus; Ab, antibody; AKT, protein kinase B; AMPK, AMP-activated protein kinase; antag., antagonist; Arg1, arginase-1; BDNF, brain-derived neurotrophic factor; bil, bilateral; BL25, Dachangshu; BL40, Weizhong; BL60, Kunlun; BV2, murine microglial cell line; BzATP, 2′(3′)-O-(4-benzoylbenzoyl)ATP; Ca^2+^, calcium ion; caspase-1, cysteine-aspartic protease 1; CCI, chronic constriction injury; CCL2, C-C motif chemokine ligand 2; CCR2, C-C motif chemokine receptor 2; CD, cluster of differentiation; CIPN, chemotherapy-induced peripheral neuropathy; contra, contralateral; COX-2, cyclooxygenase-2; CTAP, D-Phe-Cys-Tyr-D-Trp-Arg-Thr-Pen-Thr-NH_2_; CX3CR1, CX3C chemokine receptor 1; d, day(s); DAP12, DNAX-activating protein of 12 kDa; DNP, diabetic neuropathic pain; DRG, dorsal root ganglion; EA, electroacupuncture; ELISA, enzyme-linked immunosorbent assay; ephys, electrophysiology; ERK1/2, extracellular signal-regulated kinase 1/2; EX-B2, Jiaji (Huatuojiaji); F, female; Freq, stimulation frequency; GABAᴀ, γ-aminobutyric acid type A receptor; gain-of-fn, gain-of-function; GB30, Huantiao; GB34, Yanglingquan; GFAP, glial fibrillary acidic protein; GLP-1/GLP-1R, glucagon-like peptide-1 and glucagon-like peptide-1 receptor; GRK2, G protein-coupled receptor kinase 2; GV20, Baihui; HMGB1, high-mobility group box 1; Hz, hertz; Iba1, ionized calcium-binding adapter molecule 1; IENF, intraepidermal nerve fiber, IF, immunofluorescence; IFN-γ, interferon-γ; IHC, immunohistochemistry; IL, interleukin; inhib., inhibitor; iNOS, inducible nitric oxide synthase; ipsi, ipsilateral; IRF8, interferon regulatory factor 8; i.t., intrathecal; JNK, c-Jun N-terminal kinase; KD, knockdown; L3–L6, lumbar spinal segments 3–6; LC3, microtubule-associated protein 1 light chain 3; LTP, long-term potentiation; M, male; mA, milliampere; MAPK, mitogen-activated protein kinase; mech, mechanical; min, minute(s); mTOR, mammalian target of rapamycin; MWL, mechanical withdrawal latency; MWT, mechanical withdrawal threshold; MyD88, myeloid differentiation primary response 88; N-GSDMD, N-terminal gasdermin D; NF-κB, nuclear factor kappa B; NLRP3, NLR family pyrin domain containing 3; ns, not significant; OE, overexpression; OX-42, CD11b/c microglial marker; p-, phosphorylated; p62, sequestosome-1; P2X4R/P2X7R, P2X4 and P2X7 purinergic receptors; PD-1, programmed cell death protein 1; PD-L1, programmed death-ligand 1; PDYN, prodynorphin; PENK, proenkephalin; PI3K, phosphoinositide 3-kinase; PNOC, prepronociceptin; POMC, pro-opiomelanocortin; PT, pain threshold; PTX, paclitaxel; PWL, paw withdrawal latency; PWT, paw withdrawal threshold; q.o.d, every other day; RT-qPCR, reverse-transcription quantitative polymerase chain reaction; SD, Sprague–Dawley; SDH, spinal dorsal horn; sEPSC, spontaneous excitatory postsynaptic current; siRNA, small interfering RNA; SNI, spared nerve injury; SNL, spinal nerve ligation; SP6, Sanyinjiao; ST36, Zusanli; STZ, streptozotocin; TEM, transmission electron microscopy; TLR4, Toll-like receptor 4; TMEM119, transmembrane protein 119; TNF-α, tumor necrosis factor-α; TREM2, triggering receptor expressed on myeloid cells 2; TrkB, tropomyosin receptor kinase B; TRPV1, transient receptor potential vanilloid 1; TWL, thermal withdrawal latency; WB, Western blot; α7nAChR, α7 nicotinic acetylcholine receptor; β-endorphin, beta-endorphin. “Functional evidence” indicates whether an antagonist, inhibitor, antibody or antiserum, receptor blockade, or gene knockdown or overexpression was used to test the functional role of the target, as opposed to a change in expression (marker) alone.

**Table 2:** Appraisal of mechanistic evidence by pathway.

Mechanistic Category	Pathway/Target (Refs)	Number of Studies	Microglial Markers (Direction, Method, Level)	Cytokine and Mediator Data	Behavioral Outcome	Type of Functional Evidence	Predominant Risk-of-Bias Concern
1. Initiation and purinergic activation	P2X4R/BDNF [72,73,74,75]	4	↓P2X4R/OX-42 (IHC, L4–L5) [72]; ↓P2X4R/Iba1 (IF, L4–L5) [73]; ↓CD11b (IF) and ↓Iba1 (WB) [74]; ↓Iba1 (IF/WB/RT-qPCR, L4–L6) [75]	↓IFN-γ [72]; ↓TNF-α, ↓IL-1β [74]; ↓BDNF [74,75]; ↑GABAᴀγ2, LTP not induced [75]; ↓sEPSC frequency [73]	↑PWT [72]; ↑MWT [75], ↑TWL [73]; ↑PWL [74] (4/4 studies improved)	5-BDBD (P2X4R antagonist) [74,75]; IFN-γ challenge, gain-of-function [72]; none in [73]	High: blinding; unclear sequence generation and allocation
1. Initiation and purinergic activation	BDNF–TrkB [76]	1	↓BDNF/Iba1 (IF, L4–L6); ↓Iba1 (RT-qPCR, L4–L6)	↓BDNF, ↓TrkB, ↓p-TrkB; no cytokine panel reported	↑MWT, ↑TWL	None—expression change only	High: blinding; unclear allocation
1. Initiation and purinergic activation	P2X7R/p38 MAPK [77,78]	2	↓P2X7R/OX-42 (IF, L4–L5) [77]; ↓Iba1 (WB + IF, L4–L6) [78]	↓IL-1β, ↓IL-18 [77]; ↓IL-1β, ↓IL-6, ↓TNF-α, ↑IL-10,↓BDNF, ↓p-p38 MAPK [78]	↑PWT [77]; ↑MWT, ↑TWL [78]	A-438079 (P2X7R antagonist) plus cultured microglia [77]; BzATP challenge, gain-of-function [78]	High: blinding; otherwise low to unclear
1. Initiation and purinergic activation	IRF8/CX3CR1 [71]	1	↓IRF8/CD11b, ↓CX3CR1/CD11b (IF, L4–L6)	Not reported	↑PWT	None—expression change only	High: blinding; unclear attrition
2. Innate immune sensing and inflammasome	CCL2/CCR2 [79]	1	↓Iba1 (IF, L4–L6)	↓CCL2, ↓CCR2, ↓IL-1β, ↓TNF-α	↑PWT, ↑PWL	Intrathecal recombinant CCL2 challenge	High: blinding
2. Innate immune sensing and inflammasome	TLR4/MyD88/NF-κB, including HMGB1 and TRPV1 [80,81,82]	3	↓TMEM119 (WB/ELISA, lumbar cord) [80]; ↓OX-42 (IF, L4–L6 DRG and cord) [81]; ↓TLR4/CD11b, ↓MyD88/CD11b (IF, L4–L6) [82]	↓TLR4, ↓NF-κB p65, ↓IL-1β, ↓TNF-α, ↓GFAP [80]; ↓TLR4, ↓MyD88, ↓TRPV1, ↓GFAP [81]; ↓HMGB1, ↓TLR4, ↓MyD88, ↓NF-κB p65 and ↓p-NF-κB p65 [82]	↑MWL [80]; ↑PWT, ↑PWL [81]; ↑PWT and improved gait [82]	AMG9810/capsaicin (TRPV1) [81] only; none for TLR4 itself [80,82]	High: blinding; attrition concern [82]
2. Innate immune sensing and inflammasome	NLRP3 inflammasome/microglial pyroptosis [83]	1	↓NLRP3/Iba1, ↓caspase-1/Iba1, ↓N-GSDMD/Iba1 (IF, L4–L6)	↓NLRP3, ↓cleaved caspase-1, ↓N-GSDMD, ↓IL-18, ↓IL-1β, ↓TNF-α	↑MWT, ↑PWL	MCC950 (NLRP3 inhibitor)	High: blinding; otherwise low
3. Downstream amplification and neuron–glia sensitization	PI3K/AKT [84]	1	↓Iba1 (WB, L4–L5)	↓p-PI3K, ↓p-AKT, ↓TNF-α, ↓IL-6	↑PWT, ↑TWL	LY294002 (PI3K inhibitor)	High: blinding
3. Downstream amplification and neuron–glia sensitization	p38 MAPK [85]	1	↓p-p38 MAPK/OX-42 (double IF, L4–L6 SDH)	↓p-p38 MAPK; cytokines not reported	↑PWT	SB203580 (p38 inhibitor)	High: blinding
3. Downstream amplification and neuron–glia sensitization	AMPK/ mTOR–autophagy [86]	1	CD11b (IF, lumbar SDH); p62/CD11b co-localisation; ↑autophagosomes (TEM)	↑p-AMPK/AMPK, ↓p-mTOR/mTOR, ↓p62, ↑Beclin-1, ↑LC3-II/LC3-I; cytokines not reported	↑PWT	Compound C (AMPK inhibitor)	High: blinding
3. Downstream amplification and neuron–glia sensitization	COX-2 [87]	1	↓COX-2/OX-42 (IF, L4–L6)	↓COX-2; cytokines not reported	↑TWL	None—expression change only (EA-point variants compared)	High: blinding
3. Downstream amplification and neuron–glia sensitization	Microglial (early) to astrocytic (late) time course [68,69]	2	↓OX-42 (IF, L4–L6 SDH, day 3) [68]; ↓Iba1 (IF, L4–L5 SDH, 2 days after EA) [69]	↓GFAP days 3–14 [68]; ↓GFAP 1–2 weeks after EA [69]; IL-1β and IL-6 not significantly changed [68]	↑PWT [68]; ↑MWT, ↑TWL [69]	None—expression change only	High: blinding; unit-of-analysis issue [68]
4. Pro-resolution and neuroimmune modulation	IL-10/β-endorphin [88]	1	↑IL-10/Iba1, ↑β-endorphin/Iba1 (IF, L3–L5 SDH)	↑IL-10, ↑β-endorphin, ↑POMC, ↑PNOC, ↑PENK; PDYN unchanged	↑PWT	IL-10 antibody, β-endorphin antiserum, CTAP (μ-opioid antagonist)	High: blinding; low to unclear elsewhere
4. Pro-resolution and neuroimmune modulation	PD-L1/PD-1; microglia phenotypes transition [89]	1	↓Iba1; ↑Arg-1, ↑CD206; ↓CD16, ↓iNOS (WB + IF, L4–L6)	↑PD-L1, ↑PD-1; ↑IL-10; ↓IL-1β, ↓IL-6, ↓TNF-α; ↓p-p38, ↓p-ERK1/2, ↓p-JNK, ↓p-NF-κB	↑MWT, ↑TWL	PD-L1 siRNA knockdown; BV2 microglia	High: blinding
4. Pro-resolution and neuroimmune modulation	GRK2/TREM2–DAP12 [90]	1	↓CD16 (RT-qPCR, L4–L6)	↑GRK2; ↓TREM2, ↓IENF loss, ↓DAP12; ↓IL-1β, ↓IL-6, ↓iNOS	↑PWT; ↓adhesive removal time;	Neuronal GRK2 overexpression/knockdown (AAV)	High: blinding
4. Pro-resolution and neuroimmune modulation	miR-124/TREM2–DAP12 [91]	1	↓CD16/32, ↓iNOS; CD206 not significant (qPCR, SDH)	↓IENF loss, ↑miR-124; ↓TREM2, ↓DAP12; ↑IL-10; ↓TNF-α, ↓IL-1β	↑PWT; ↓adhesive removal time;	Neuronal miR-124 overexpression/knockdown (AAV)	High: blinding
4. Pro-resolution and neuroimmune modulation	α7nAChR [92]	1	↑α7nAChR, ↓CD11b (IF, L4–L6 SDH)	↑α7nAChR; ↓IL-1β	↑PWT	α-bungarotoxin (α7nAChR antagonist)	High: blinding
4. Pro-resolution and neuroimmune modulation	GLP-1/GLP-1R [93]	1	↓Iba1 (IF + WB, L4–L6 SDH)	↑GLP-1, ↑GLP-1R; ↓GFAP; cytokines not reported	↑PWT, ↑TWL	Exendin-4 (agonist)/exendin-(9–39) (antagonist)	High: blinding
4. Pro-resolution and neuroimmune modulation	GABAᴀ receptors [94]	1	↓Iba1 (WB + IF, L4–L6)	↑GABAᴀ α2, α3 and γ2 subunits; cytokines not reported	↑MWT, ↑TWL	Bicuculline (GABAᴀ antagonist)	High: blinding

Arrows indicate the direction of change in the electroacupuncture group relative to the untreated model group. In the microglial-marker and cytokine-and-mediator columns, ↓ denotes a decrease and ↑ an increase in expression, phosphorylation, or immunoreactivity. In the behavioral-outcome column, the arrow denotes the direction of the measured variable rather than the severity of pain: ↑PWT, ↑MWT, ↑PWL, ↑TWL and ↑MWL (increased withdrawal thresholds or latencies), and ↓adhesive removal time, all indicate attenuation of pain-related hypersensitivity or neuropathy. 5-BDBD, 5-(3-bromophenyl)-1,3-dihydro-2H-benzofuro [3,2-e]-1,4-diazepin-2-one; AAV, adeno-associated virus; AKT, protein kinase B; AMPK, AMP-activated protein kinase; Arg-1, arginase-1; BDNF, brain-derived neurotrophic factor; Beclin-1, coiled-coil myosin-like BCL2-interacting protein; BV2, murine microglial cell line; BzATP, 2′(3′)-O-(4-benzoylbenzoyl)ATP; caspase-1, cysteine-aspartic protease 1; CCL, C-C motif chemokine ligand; CCR, C-C motif chemokine receptor; CD, cluster of differentiation; COX-2, cyclooxygenase-2; CTAP, D-Phe-Cys-Tyr-D-Trp-Arg-Thr-Pen-Thr-NH_2_; CX3CR1, CX3C chemokine receptor 1; DAP12, DNAX-activating protein of 12 kDa; DRG, dorsal root ganglion; EA, electroacupuncture; ERK1/2, extracellular signal-regulated kinase 1/2; GABAᴀ, γ-aminobutyric acid type A receptor; GFAP, glial fibrillary acidic protein; GLP-1/GLP-1R, glucagon-like peptide-1 and glucagon-like peptide-1 receptor; GRK2, G protein-coupled receptor kinase 2; HMGB1, high-mobility group box 1; Iba1, ionized calcium-binding adapter molecule 1; IENF, intraepidermal nerve fiber; IF, immunofluorescence; IFN-γ, interferon-γ; IHC, immunohistochemistry; IL, interleukin; iNOS, inducible nitric oxide synthase; IRF8, interferon regulatory factor 8; JNK, c-Jun N-terminal kinase; L3–L6, lumbar spinal segments 3–6; LC3, microtubule-associated protein 1 light chain 3; LTP, long-term potentiation; MAPK, mitogen-activated protein kinase; miR-124, microRNA-124; mTOR, mammalian target of rapamycin; MWL, mechanical withdrawal latency; MWT, mechanical withdrawal threshold; MyD88, myeloid differentiation primary response 88; N-GSDMD, N-terminal gasdermin D; NF-κB, nuclear factor kappa B; NLRP3, NLR family pyrin domain containing 3; OX-42, CD11b/c microglial marker; p-, phosphorylated; p62, sequestosome-1; P2X4R/P2X7R, P2X4 and P2X7 purinergic receptors; PD-1, programmed cell death protein 1; PD-L1, programmed death-ligand 1; PDYN, prodynorphin; PENK, proenkephalin; PI3K, phosphoinositide 3-kinase; PNOC, prepronociceptin; POMC, pro-opiomelanocortin; PWL, paw withdrawal latency; PWT, paw withdrawal threshold; qPCR, quantitative polymerase chain reaction; RT-qPCR, reverse-transcription quantitative polymerase chain reaction; SDH, spinal dorsal horn; sEPSC, spontaneous excitatory postsynaptic current; siRNA, small interfering RNA; TEM, transmission electron microscopy; TLR4, Toll-like receptor 4; TMEM119, transmembrane protein 119; TNF-α, tumor necrosis factor-α; TREM2, triggering receptor expressed on myeloid cells 2; TrkB, tropomyosin receptor kinase B; TRPV1, transient receptor potential vanilloid 1; TWL, thermal withdrawal latency; WB, Western blot; α7nAChR, α7 nicotinic acetylcholine receptor. “Type of functional evidence” indicates whether the target’s functional role was tested by loss-of-function manipulation (receptor antagonist, pharmacological inhibitor, antibody or antiserum blockade, gene knockdown) or gain-of-function manipulation (agonist challenge, pathway activation, overexpression), as opposed to being supported by a change in expression (marker) alone.

## Data Availability

No new data were created or analyzed in this study.

## Supplementary Materials

The following supporting information can be downloaded at: https://www.mdpi.com/article/10.3390/brainsci16090914/s1. Table S1: The complete database-specific strategies; Table S2: SYRCLE assessment.

## Abbreviations

The following abbreviations are used in this manuscript:

Akt	Protein kinase B
AMPAR	Alpha-amino-3-hydroxy-5-methyl-4-isoxazolepropionic acid receptor
BDNF	Brain-derived neurotrophic factor
CCI	Chronic Constriction Injury
CCL/CCR	C-C motif chemokine ligand and C-C chemokine receptor
COX-2	Cyclooxygenase-2
CSF1/CSF1R	Colony-stimulating factor 1 and colony-stimulating factor 1 receptor
DAP12	DNAX-activating protein of 12 kDa
EA	Electroacupuncture
FKN	Fractalkine
GABA/GABAᴀ	γ-aminobutyric acid and γ-aminobutyric acid type A receptor
GFAP	Glial fibrillary acidic protein
GLP-1/GLP-1R	Glucagon-like peptide-1 and glucagon-like peptide-1 receptor
GRK2	G protein-coupled receptor kinase 2
HMGB1	High mobility group box 1
HSPs	Heat shock proteins
IASP	International Association for the Study of Pain
Iba1	Ionized calcium-binding adapter molecule 1
IL-1β/IL-6/IL-10/IL-18	Interleukin-1β, interleukin-6, interleukin-10, and interleukin-18
iNOS	Inducible nitric oxide synthase
IRF8	Interferon Regulatory Factor 8
KCC2	Potassium-chloride cotransporter 2
MAPK	Mitogen-activated protein kinase
MiR-124	MicroRNA-124
MyD88	Myeloid differentiation primary response 88
NF-κB	Nuclear factor kappa B
NLRP3	NOD-like receptor family pyrin domain containing 3
NMDA	N-methyl-D-aspartate
NO	Nitric oxide
NP	Neuropathic pain
P2X4R/P2X7R/P2Y12R	P2X4, P2X7, and P2Y12 purinergic receptors
PD-1/PD-L1	Programmed cell death protein 1 and programmed death-ligand 1
ROS	Reactive Oxygen Species
SDH	Spinal Dorsal Horn
sEPSCs	Spontaneous excitatory postsynaptic currents
SNI	Spared Nerve Injury
SNL	Spinal Nerve Ligation
TLR4	Toll-like receptor 4
TNF-α	Tumor necrosis factor-α
TREM2	Triggering receptor expressed on myeloid cells 2
α7nAChR	Alpha 7 nicotinic acetylcholine receptor
