# Supplementary material for: Molecular Mechanisms of Electroacupuncture-Induced Spinal Microglial Reprogramming in Neuropathic Pain: A Systematic Search–Narrative Review
Source: Brain Sci. 2026 Aug 27;16(9):914. doi: 10.3390/brainsci16090914 (PMC13604875; doi:10.3390/brainsci16090914)
Supplement: Supplementary file 1 [file brainsci-16-00914-s001.zip › Supplementary Table S1.pdf]

Supplementary Table: S1

Databases	Search Strategies
Pubmed/Medline	(("Electroacupuncture"[MeSH Terms] OR electroacupuncture[tiab] OR "electro-acupuncture"[tiab] OR "electro acupuncture"[tiab]))<br>AND<br>(("Microglia"[MeSH Terms] OR microglia[tiab] OR "microglial activation"[tiab] OR "activated microglia"[tiab] OR "microglial polarization"[tiab] OR neuroinflammation[tiab]))<br>AND<br>(("Neuropathic Pain"[MeSH Terms] OR neuralgia[MeSH Terms] OR "neuropathic pain"[tiab] OR neuralgia[tiab] OR "nerve injury"[tiab] OR "peripheral neuropathy"[tiab]))
Web of Science	#1 TS=(electroacupuncture OR "electro-acupuncture" OR "electro acupuncture")<br>#2 TS=(microglia OR "microglial activation" OR "activated microglia" OR "microglial polarization" OR neuroinflammation)<br>#3 TS=("neuropathic pain" OR neuralgia OR "nerve injury" OR "peripheral neuropathy")<br>#4 #1 AND #2 AND #3
CINAHL	S1: (MH "Electroacupuncture") OR (TI electroacupuncture OR AB electroacupuncture OR TI "electro-acupuncture" OR AB "electro-acupuncture" OR TI "electro acupuncture" OR AB "electro acupuncture")<br>S2: (MH "Microglia") OR (TI microglia OR AB microglia OR TI "microglial activation" OR AB "microglial activation" OR TI "activated microglia" OR AB "activated microglia" OR TI "microglial polarization" OR AB "microglial polarization" OR TI neuroinflammation OR AB neuroinflammation)<br>S3: (MH "Neuropathic Pain") OR (MH "Neuralgia") OR (TI "neuropathic pain" OR AB "neuropathic pain" OR TI neuralgia OR AB neuralgia OR TI "nerve injury" OR AB "nerve injury" OR TI "peripheral neuropathy" OR AB "peripheral neuropathy")<br>S4: S1 AND S2 AND S3
Cochrane	#1 [MeSH descriptor: Electroacupuncture] OR (electroacupuncture OR "electro-acupuncture" OR "electro acupuncture"):ti,ab,kw<br>#2 [MeSH descriptor: Microglia] OR (microglia OR "microglial activation" OR "activated microglia" OR "microglial polarization" OR neuroinflammation):ti,ab,kw<br>#3 [MeSH descriptor: Neuropathic Pain] OR [MeSH descriptor: Neuralgia] OR ("neuropathic pain" OR neuralgia OR "nerve injury" OR "peripheral neuropathy"):ti,ab,kw<br>#4 #1 AND #2 AND #3
